# Human preprocalcitonin self-antigen generates TAP-dependent and -independent epitopes triggering optimised T-cell responses toward immune-escaped tumours

**DOI:** 10.1038/s41467-018-07603-1

**Published:** 2018-11-30

**Authors:** Aurélie Durgeau, Yasemin Virk, Gwendoline Gros, Elodie Voilin, Stéphanie Corgnac, Fayçal Djenidi, Jérôme Salmon, Julien Adam, Vincent de Montpréville, Pierre Validire, Soldano Ferrone, Salem Chouaib, Alexander Eggermont, Jean-Charles Soria, François Lemonnier, Eric Tartour, Nathalie Chaput, Benjamin Besse, Fathia Mami-Chouaib

**Affiliations:** 1 INSERM UMR 1186, Integrative Tumour Immunology and Genetic Oncology, Gustave Roussy, EPHE, PSL, Fac. de Médecine, Univ. Paris-Sud, Université Paris-Saclay, 94805 Villejuif, France; 2 ElyssaMed, Paris Biotech Santé, 75014 Paris, France; 3 CNRS (Centre National de la Recherche Scientifique) UMR 8122, Gustave Roussy, Faculté de Médecine, Univ. Paris-Sud, Université Paris-Saclay, 94805 Villejuif, France; 4 INSERM U 981, Gustave Roussy, Faculté de Médecine, Univ. Paris-Sud, Université Paris-Saclay, 94805 Villejuif, France; 5 Service d’Anatomie Pathologique, Centre Chirurgical Marie-Lannelongue, 92350 Le-Plessis-Robinson, France; 6 Service d’Anatomie Pathologique, Institut Mutualiste Montsouris, 75014 Paris, France; 7 Department of Surgery, Massachusetts General Hospital, Harvard Medical School, Boston, MA 02114 USA; 8 Cancer Institute, Gustave Roussy Cancer Campus, Grand Paris, 94805 Villejuif, France; 9 Department of Drug Development (DITEP), Gustave Roussy, 94805 Villejuif, France; 10 Département Endocrinologie, Métabolisme et Diabète, Equipe Immunologie des Diabètes, INSERM U1016, 75014 Paris, France; 11 INSERM U970, Paris Cardiovascular Research Centre, Université Paris-Descartes, Sorbonne Paris Cité, Equipe Labellisée Ligue Contre le Cancer, Hôpital Européen Georges Pompidou, Service d’Immunologie Biologique, 75015 Paris, France; 12 Laboratory of Immunomonitoring in Oncology, and CNRS-UMS 3655 and INSERM-US23, Gustave Roussy Cancer Campus, Villejuif, France; 13 Faculté de Pharmacie, University Paris-Sud, F-92296 Chatenay-Malabry, France; 14 Département de Médecine, Gustave Roussy, 94805 Villejuif, France; 15 Present Address: Thumbay Institute for Precision Medicine, Gulf Medical University, Ajman, 4184 UAE

## Abstract

Tumours often evade CD8 T-cell immunity by downregulating TAP. T-cell epitopes associated with impaired peptide processing are immunogenic non-mutated neoantigens that emerge during tumour immune evasion. The preprocalcitonin (ppCT)_16–25_ neoepitope belongs to this category of antigens. Here we show that most human lung tumours display altered expression of TAP and frequently express ppCT self-antigen. We also show that ppCT includes HLA-A2-restricted epitopes that are processed by TAP-independent and -dependent pathways. Processing occurs in either the endoplasmic reticulum, by signal peptidase and signal peptide peptidase, or in the cytosol after release of a signal peptide precursor or retrotranslocation of a procalcitonin substrate by endoplasmic-reticulum-associated degradation. Remarkably, ppCT peptide-based immunotherapy induces efficient T-cell responses toward antigen processing and presenting machinery-impaired tumours transplanted into HLA-A*0201-transgenic mice and in NOD-*scid-Il2rγ*^*null*^ mice adoptively transferred with human PBMC. Thus, ppCT-specific T lymphocytes are promising effectors for treatment of tumours that have escaped immune recognition.

## Abstract (web summary)

Tumours can escape CD8 T-cell immunity by down-regulating antigen presentation machinery components, such as TAP. Here the authors describe tumour antigenic peptides processed by TAP-independent and -dependent pathways and show in mouse models that these peptides can be exploited to induce antitumor T-cell activity when TAP expression is downregulated.

## Introduction

Cytotoxic T lymphocytes (CTLs) are the major effectors of the immune system capable of eliminating transformed cells following recognition, by the T cell receptor (TCR), of specific antigenic peptides presented by the major histocompatibility complex class I (MHC-I)–beta-2-microglobulin (β2m) complex. Therefore, immunotherapy strategies have been developed to induce a strong persistent antitumour CTL response in order to destroy primary cancer cells and metastases. Current immunotherapies consist of stimulating tumour-specific T cells via therapeutic vaccination of cancer patients with tumour-associated antigens (TAA) or adoptively transferring in vitro expanded native or engineered T lymphocytes targeting malignant cells^1,2^. Moreover, identification of T cell surface molecules such as CTL-associated antigen-4 (CTLA-4) and programmed death-1 (PD-1), involved in regulation of antigen-specific T cell responses, has recently led to the development of promising new immunotherapies against cancer^3–6^. Indeed, treatment of cancer patients with neutralizing monoclonal antibodies (mAbs) specific to these T cell inhibitory receptors has resulted in impressive response rates and, in some cases, durable remission, emphasizing the central role of endogenous T lymphocytes in defence against malignant cells. In this context, it has been reported that tumour regression following therapeutic PD-1 blockade requires pre-existing CD8^+^ T lymphocytes that are negatively regulated by PD-1/PD-ligand 1 (PD-L1)-mediated adaptive immune resistance^7^. More recent studies demonstrated that T cell reactivity towards tumour-specific mutated antigens, called neoantigens, is directly associated with clinical benefits of adoptive T cell therapy, immune checkpoint blockade and peptide-based cancer vaccines^8–17^. This implies that, in responding patients, endogenous T lymphocytes are able to recognize peptide neoepitopes displayed on the surface of malignant cells by MHC molecules and to trigger antitumour immune responses.

Unfortunately, only a fraction of cancer patients respond to these T cell-based therapeutic interventions, indicating that multiple additional mechanisms leading to tumour resistance to immunotherapy exist. In this context, it was recently demonstrated that patients identified as non-responders to anti-CTLA-4 mAbs have tumours with genomic defects in interferon (IFN)-γ pathway genes^18^. Moreover, primary or acquired resistance to PD-1 blockade immunotherapy was associated with defects in pathways involved in IFN-γ-receptor signalling and antigen presentation by MHC-I molecules^19,20^. Among additional known mechanisms involved in tumour resistance to T cell-mediated immunity, alterations in antigen processing play an important role. Indeed, accumulating evidence indicates that defects in transporter associated with antigen processing (TAP) subunits result in a sharp decrease in surface expression of MHC-I/peptide complexes, enabling escape of malignant cells from CD8 T cell recognition. In this regard, it was recently reported that T lymphocytes specific to a non-mutated self-epitope derived from the C-terminus region of the TRH4 protein, defined as a T cell epitope associated with impaired peptide processing (TEIPP), were efficiently selected in the thymus of TCR transgenic mice and might be activated by peptide-based vaccination, leading to growth control of TAP-deficient tumours expressing low levels of MHC-I/peptide complexes^21^. In humans, we had previously identified a non-mutated tumour epitope derived from the preprocalcitonin (ppCT) signal peptide (ppCT_16–25_) by a mechanism independent of proteasomes and TAP, involving signal peptidase (SP) and signal peptide peptidase (SPP)^22^. In this report, we define three additional HLA-A2-restricted T cell epitopes derived from either the hydrophobic core region (h-region) of the ppCT signal peptide (ppCT_9–17_) or the procalcitonin (pCT) precursor protein (ppCT_50–59_ and ppCT_91–100_). They are processed in the cytosol after release of a peptide precursor from the ppCT leader sequence by SPP or after retrotranslocation of a pCT substrate from the endoplasmic reticulum (ER) lumen by the ER-associated degradation (ERAD) pathway, respectively. Importantly, active immunotherapy based on a cocktail of five ppCT peptides, including ppCT_16–25_, ppCT_9–17_ and a 15-amino acid (aa)-long peptide derived from the NH2-terminal region of the ppCT leader sequence (ppCT_1–15_), was able to induce antitumour CTL responses in HLA-A*0201/HLA-DR3-transgenic (HHD-DR3) mice and NOD*-scid-Il2rγ*^*null*^ (NSG) mice adoptively transferred with human peripheral blood mononuclear cells (PBMCs), capable of controlling growth of established tumours expressing low levels of HLA-A2/human ppCT peptide complexes. We propose that ppCT leader sequence-derived peptides constitute promising T cell targets permitting CTL to eradicate tumours with impaired antigen processing and presenting machinery (APM) and thus overcome tumour escape from CD8 T cell immunity.

## Results

### ppCT and TAP expression in human lung tumours

To further extend our previous studies^22^ on the prevalence of *CALCA* gene expression in primary human lung tumours, we first evaluated the level of the *calcitonin* (*CT*) transcript in tumours from 28 additional non-small-cell lung carcinoma (NSCLC) patients and allogeneic normal thyroids, used as a reference, by quantitative real-time PCR (qRT-PCR). High expression levels of *CT* mRNA were detected in several lung cancer samples as compared to allogeneic thyroid tissues (Table 1). Indeed, up to 39% of lung tumour tissues, mainly from adenocarcinoma (ADC) histological subtypes, (over)expressed the *CT* transcript, with levels ranging from 2- to 2,000-fold higher than those found in normal human thyroids. We then confirmed the expression of CT at the protein level by immunohistochemistry (IHC) in a cohort of 215 formalin-fixed paraffin-embedded (FFPE) lung tumour samples (Supplementary Figure 1a), where up to 20% of ADC and 38% of neuroendocrine tumours (NET) expressed the protein (Table 2).

**Table 1:** Relative expression of *CT* and *TAP* transcripts in lung tumour samples

Histological type	Tumour sample	Relative expression of *CT* transcript	Relative expression of *TAP1* transcript	Relative expression of *TAP2* transcript
ADC	ADC-1	0.18	***0.26***	***0.47***
	ADC-2	1.24	1.24	1.61
	ADC-3	**92.41**	*2.18*	*2.71*
	ADC-4	**2112.89**	***0.44***	0.63
	ADC-5	**13.32**	2.03	0.9
	ADC-6	**2.11**	0.57	***0.46***
	ADC-7	**368.37**	***0.07***	***0.08***
	ADC-8	1.69	2.68	1.34
	ADC-9	**5.96**	***0.29***	***0***
	ADC-10	0.27	0.75	***0.48***
	ADC-11	**652.58**	0.74	0.93
	ADC-12	0.53	***0.08***	***0.1***
	ADC-13	**3.68**	0.53	***0.15***
	ADC-14	0.41	1.46	2.53
LCC	LCC-1	0.65	***0***	***0.06***
	LCC-2	1.74	1.98	0.76
	LCC-3	0.17	***0.41***	0.65
	LCC-4	0	***0.21***	***0.3***
	LCC-5	1.27	***0***	***0***
SCC	SCC-1	**747.02**	7.26	8
	SCC-2	**3.13**	***0.18***	***0.16***
	SCC-3	**4.16**	***0***	***0***
	SCC-4	0.64	***0.17***	***0.27***
	SCC-5	0.2	***0.03***	***0.01***
	SCC-6	0	***0.05***	***0.11***
NET	NET-1	0.29	***0.05***	***0.05***
	NET-2	0.84	***0.33***	***0.42***
	NET-3	0.17	***0.03***	***0.09***

qRT-PCR analysis of *CT* and *TAP* transcripts in fresh human lung tumour samples. Normalized copy numbers of *CT* and *TAP* transcripts are shown. The expression of *CT* was normalized to allogeneic healthy thyroid tissues, and expression of *TAP* was normalized to autologous healthy lung tissue. Values of the *CT* transcript that are statistically elevated are shown in bold and those of *TAP* that are statistically downregulated are shown in bold and italics (*P* < 0.001 according to the Mann–Whitney *U* test)

*NET* neuroendocrine tumours, *ADC* adenocarcinomas, *LCC* large cell carcinomas, *SCC* squamous cell carcinomas

**Table 2:** Expression of CT protein in lung tumours

Histological type	Low	Medium	High	Percentage
ADC (67 samples)	5	7	1	20%
SCC (35 samples)	0	0	0	0%
NET (58 samples)	7	6	9	38%
Undif (47 samples)	2	2	0	9%
Other (8 samples)	0	1	1	25%
Total	14/215	16/215	11/215	19%/215

Calcitonin (CT) protein expression in a cohort of 215 FFPE lung tumour samples was performed by IHC

*NET* neuroendocrine tumours, *ADC* adenocarcinomas, *SCC* squamous cell carcinomas, *Undif* undifferentiated

Our previous studies had demonstrated that downregulation of TAP1 or TAP2 subunits potentiates ppCT_16–25_ epitope presentation on tumour cells expressing the *CALCA* gene^23^. We therefore evaluated the prevalence of TAP downregulation in human lung cancer specimens by analysing the expression levels of *TAP1* and *TAP2* mRNA in primary human tumours and autologous normal lungs. qRT-PCR studies indicated that up to 71% of the 28 analysed lung tumours expressed low levels of *TAP1* and/or *TAP2* mRNA as compared to autologous normal lungs (Table 1). To estimate the percentage of tumours with TAP protein defects, we performed IHC staining with anti-TAP2 mAb in a cohort of 135 FFPE lung tumour samples (Supplementary Figure 1b). Results indicated that 53% and 32% of the tumours displayed low and intermediate expression levels of TAP2, respectively, while only 14% of human lung tumours expressed high levels of the TAP2 subunit (Table 3). These results suggest that immunotherapy based on the ppCT precursor protein may help to overcome tumour escape from CD8 T cell immunity associated with TAP subunit expression defects.

**Table 3:** Expression of TAP2 protein in lung tumours

Histological type	Low	Intermediate	High
ADC (56 samples)	22/56 (39%)	25/56 (45%)	9/56 (16%)
SCC (29 samples)	20/29 (69%)	7/29 (24%)	2/29 (7%)
Undif (42 samples)	24/42 (57%)	10/42 (24%)	8/42 (19%)
Other (8 samples)	6/8 (75%)	2/8 (25%)	0/8 (0%)
Total	72/135 (53%)	44/135 (32%)	19/135 (14%)

TAP2 protein expression in a cohort of 135 FFPE lung tumour samples was performed by IHC. Data correspond to TAP2 *H*-score. Low: 0 ≤ *H*-score ≤ 70; Intermediate: 80 ≤ *H*-score ≤ 140; High: 160 ≤ *H*-score ≤ 200. H-score = % TAP2^+^ cells × TAP2 intensity. TAP2 intensity varies from 0 to 2. Normal cell *H*-score = 200

*ADC* adenocarcinomas, *SCC* squamous cell carcinomas, *Undif* undifferentiated

### Selection of HLA-A2-binding peptides derived from the ppCT

Next, we asked whether ppCT includes additional HLA-A*0201-restricted epitopes that could trigger an antitumour CTL response. With this aim, we screened the entire sequence of the ppCT precursor for the presence of peptides with high binding affinity for HLA-A*0201 using the epitope prediction software SYFPEITHI. We selected two peptides, ppCT_9–17_ and ppCT_50–59_, with high predicted binding scores and derived from the hydrophobic central region (h-region) of the ppCT signal peptide and the pCT prohormone, respectively (Table 4). We also selected additional peptides, such as ppCT_5–14_, ppCT_41–49_, ppCT_53–62_, ppCT_87–96_ and ppCT_91–100_, with a predicted binding score higher than or similar to that of ppCT_16–25_ (Table 4, Supplementary Table 1).

**Table 4:** Identification of HLA-A2-restricted ppCT epitopes

Peptide	aa Sequence	SYFPEITHI prediction	FI	DC_50_ (h)
ppCT_9–17_	FLALSILVL	28	1.27	5
ppCT_16–25_	VLLQAGSLHA	18	0.24	3
ppCT_50–59_	LLAALVQDYL	24	2.58	10
ppCT_91–100_	CMLGTYTQDF	12	0.30	4
E27L Mart-1_26–35_	ELAGIGILTV	28	3.03	8

Fixation and stabilization assays were performed with 100 µM of a given peptide. FI (fluorescence index) = (mean fluorescence intensity (MFI) with the given peptide − MFI without peptide)/MFI without peptide; DC_50_: half-life of the HLA-A2–peptide complexes

*aa* amino acid

We then evaluated the capacity of all the selected peptides to bind to HLA-A*0201 and to form stable HLA-A*0201/peptide complexes using the TAP-deficient HLA-A2-transfected T2 cell line. Similarly to the mutated E27L MelanA/Mart-1_26–35_ epitope, used as a positive control^24,25^, ppCT_50–59_, ppCT_41–49_ and ppCT_9–17_ peptides and, to a much lesser extent, ppCT_91–100_, displayed high binding affinity and stabilization capacity towards HLA-A*0201, fulfilling the characteristics of immunogenic peptides^26^ (Table 4). In contrast, other peptides with high predictive scores, such as ppCT_5–14_, ppCT_53–62_ and ppCT_87–96_, were excluded because they displayed very low binding affinity towards HLA-A*0201 (Supplementary Table 1). These results suggest that the ppCT preprohormone includes at least two additional HLA-A2-restricted epitopes that may induce a CD8 T cell response.

### ppCT_9–17_, ppCT_50–59_ and ppCT_91–100_ are immunogenic epitopes

**Fig. 1:**
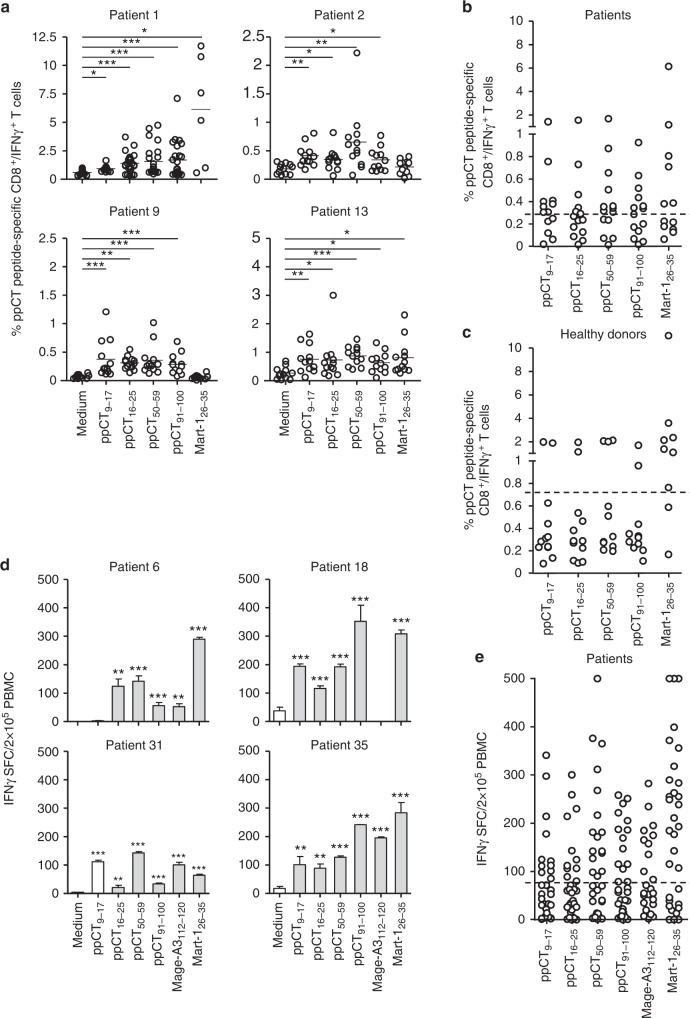
Immunogenicity of ppCT-derived epitopes in NSCLC patient PBMCs. **a** Cytoplasmic expression of IFN-γ in CD8^+^ T cells as determined by cytoplasmic immunofluorescence analysis. PBMCs were stimulated for 14 days in 96-well plates with the indicated peptides or with medium (containing DMSO alone), then restimulated for 6 h in the same conditions, as described in Methods. Cells from eight culture wells (one column) were pooled and then stained with anti-CD8 mAb, and after membrane permeabilization, they were stained with anti-IFN-γ mAb. For each peptide, 12–15 columns were included. For media and control peptides, six columns were tested for patients for whom insufficient amounts of PBMCs were available. Samples were analysed using an Accuri C6 cytometer, and data were processed by the Cflow software. Circles correspond to the percentages of positive cells in one column from a 96-well plate. Horizontal lines correspond to mean percentages of positive cells. Data shown are from 4 patients out of 15. *p<0.05; **p < 0.01; ***p < 0.001 (two-tailed Mann–Whitney *U* test). **b** Mean percentages of IFN-γ-producing CD8^+^ T cells from 15 analysed NSCLC patients’ PBMCs. The horizontal dashed line corresponds to the cutoff value (e.g. mean of the medium condition mean values plus 2 times the SD) above which responses are considered positive. **c** Mean percentages of IFN-γ-producing CD8^+^ T cells from 12 analysed healthy donors’ PBMCs. The horizontal dashed line corresponds to the cutoff value above which responses are considered positive. **d** IFN-γ SFC from NSCLC patients’ PBMCs. PBMCs from NSCLC patients were stimulated in 96-well plates with the indicated peptides (24 culture wells for each peptide) as described in **a**; then cells were pooled, and IFN-γ-secreting cells were evaluated by Elispot assay. Values correspond to means (±SD) of SFC from triplicates. Data shown are from 4 patients out of 28. **p < 0.01; ***p < 0.001 (two-tail Student’s unpaired *t* test with Welch’s correction). **e** Means of IFN-γ SFC from 28 patients’ PBMCs. The horizontal dashed line corresponds to the cutoff value above which responses are considered positive. HLA-A2-restricted Mart-1_26–35_ and/or Mage-A3_112–120_ (KVAELVHFL) peptides were included as controls. SFC spot-forming cells

To determine whether the identified ppCT peptides are immunogenic, we first analysed their capacity to activate specific human CD8^+^ T lymphocytes in vitro. For this purpose, we twice stimulated, at 1-week intervals, lung cancer patient PBMCs with each of the four peptides and then evaluated generation of ppCT-specific CTLs by intracellular IFN-γ staining or the enzyme-linked immunospot (Elispot) assay. The ppCT_41–49_ peptide was rapidly excluded because it was not immunogenic in any of the five patient and healthy donor PBMCs tested. Among HLA-A2^+^ patients tested by intracellular staining (see Supplementary Figure 2a), 8 out of 13, 10 out of 15 and 7 out of 15 patients triggered IFN-γ-producing CD8^+^ T cells towards ppCT_9–17_, ppCT_50–59_ and ppCT_91–100_ peptides, respectively (Fig. 1a, b). The ppCT_16–25_ and MelanA/Mart-1_26–35_ epitopes, included as positive controls, induced specific IFN-γ-producing CD8^+^ T cells in 5 out of 15 and 6 out of 13 patient PBMCs, respectively (Fig. 1a, b). Induction of specific IFN-γ-producing cells was also observed in some PBMCs from HLA-A2^+^ healthy donors (Supplementary Figure 1b and Fig. 1c). Moreover, Elispot assay confirmed induction of peptide-specific IFN-γ-producing cells in PBMCs isolated from several NSCLC patients among the 28 additional patients tested (Fig. 1d, e). These results indicate that lung cancer patients respond 2.5- to 3.5-fold more frequently to ppCT peptides than healthy donors (Supplementary Figure 2c).

**Fig. 2:**
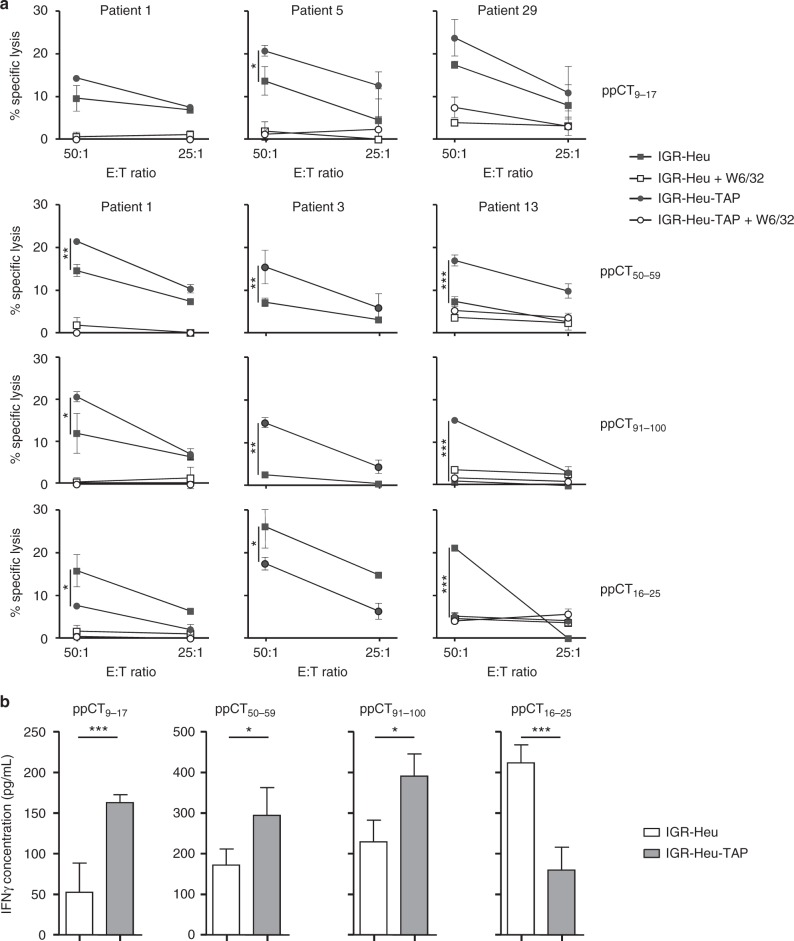
Specificity of ppCT-peptide-stimulated CD8^+^ T cells. **a** NSCLC patient PBMCs were stimulated in vitro with the indicated peptides, and then CD8^+^ T cells were isolated and their cytotoxic activity was tested. The IGR-Heu and IGR-Heu-TAP (IGR-Heu transfected with TAP1 and TAP2) tumour cell lines, generated from patient 1 (Heu), pre-incubated or not with neutralizing anti-MHC-I mAb W6/32, were used as targets. Cytotoxicity was determined by a conventional 4-h ^51^Cr release assay at the indicated E:T ratios. Values correspond to means (±SD) of percentages of lysis from triplicates. Data shown represent experiments from three patients' PBMCs. **b** Cytokine release of patient 1’s T cell clones and T cell cloids stimulated with the autologous IGR-Heu and IGR-Heu-TAP cell lines. IFN-γ release was measured using ELISA. Data shown are means (±SD) of three T cell clones and three T cell cloids from three independent experiments. *p < 0.05; ***p < 0.001 (two-tailed Student’s unpaired *t* test). E:T effector:target

We then addressed the question of whether ppCT_9–17_, ppCT_50–59_ and ppCT_91–100_ were naturally processed by tumour cells by testing the capacity of responding T cell lines to recognize the ppCT^high^TAP^low^ IGR-Heu cell line generated from patient 1 (Heu) and the IGR-Heu-TAP cell line, which we had previously transfected with TAP1/2-encoding plasmids^23^. In agreement with our previous studies^23^, CTL induced towards ppCT_16–25_ lysed the parental IGR-Heu tumour cell line more efficiently than the TAP-transfected target (Fig. 2a). In contrast, ppCT_9–17_-, ppCT_50–59_- and ppCT_91–100_-specific CTL displayed stronger cytotoxic activity towards TAP-efficient than towards TAP-deficient tumour cells. Cytotoxicity towards IGR-Heu and IGR-Heu-TAP target cells was inhibited by anti-MHC-I mAb (W6/32), indicating that it is most likely TCR mediated (Fig. 2a). Peptide specificity was further confirmed using HLA-A2^+^ Epstein–Barr virus (EBV)-transformed B cells generated from patient 1 (Heu-EBV), unpulsed or pulsed with each of the peptides (Supplementary Figure 3a). Results indicated that CD8^+^ T cells generated towards ppCT peptides more efficiently killed specific peptide-loaded B cells than unloaded B cells. In contrast, they were unable to kill the natural killer-sensitive target cell line K562, further supporting the conclusion that cytotoxicity towards IGR-Heu tumour cells is specific and TCR mediated (Supplementary Figure 3a). Moreover, cytotoxicity towards the IGR-Heu-TAP tumour cell line was inhibited by adding an excess of competing unlabelled target cells pulsed with ppCT_9–17_, ppCT_50–59_ or ppCT_91–100_ peptide (Supplementary Figure 3b). We then generated, from patient 1, several T cell cloids and T cell clones towards each of the ppCT epitopes and measured IFN-γ production upon stimulation with autologous IGR-Heu and IGR-Heu-TAP tumour cell lines. Data showed that, while T cell cloids generated towards ppCT_9–17_, ppCT_50–59_ and ppCT_91–100_ epitopes produced higher levels of IFN-γ when stimulated with IGR-Heu-TAP, T cell clones generated towards the ppCT_16–25_ epitope produced higher cytokine levels when stimulated with autologous IGR-Heu tumour cells (Fig. 2b). Notably, CTL generated towards ppCT-derived peptides also killed, with variable killing efficiency, the TAP-proficient ppCT^+^ medullary thyroid carcinoma (MTC) cell line TT (Supplementary Figure 3c). Thus ppCT_9–17_, ppCT_16–25_, ppCT_50–59_ and ppCT_91–100_ epitopes appear to be more immunogenic in NSCLC patients than in healthy donors and are naturally processed in ppCT-expressing tumour cells.

### Processing of ppCT_9–17_, ppCT_50–59_ and ppCT_91–100_ epitopes

**Fig. 3:**
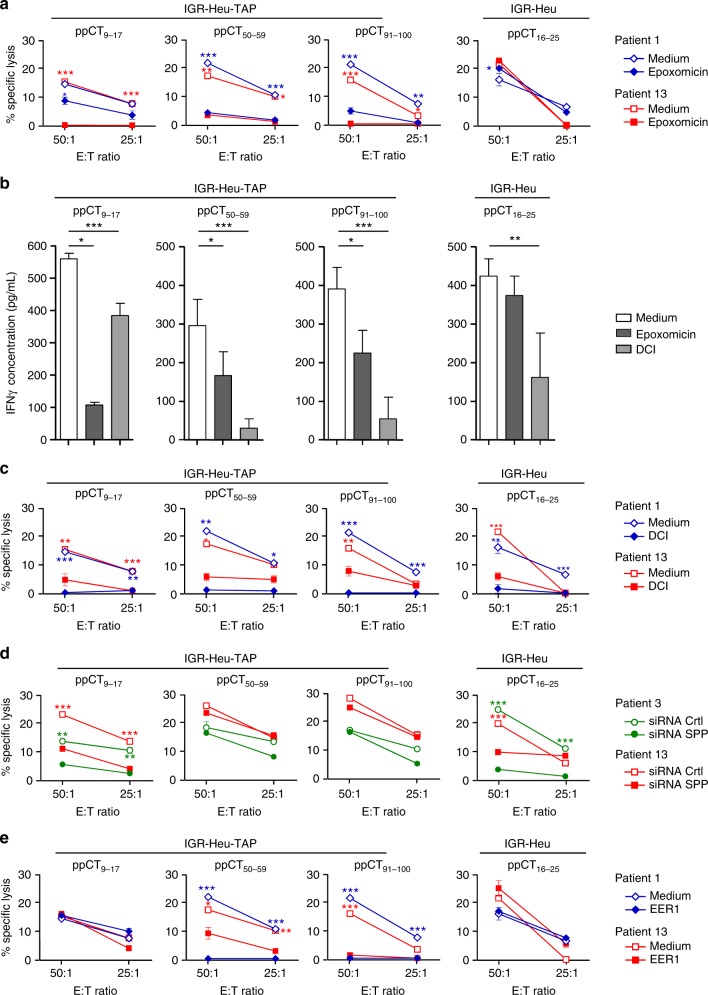
Processing pathway of the ppCT_9–17_, ppCT_50–59_ and ppCT_91–100_ epitopes. **a** Processing of pCT_9–17_, ppCT_50–59_ and ppCT_91–100_ peptides is proteasome dependent. IGR-Heu or IGR-Heu-TAP cells were incubated in the absence or presence of the proteasome inhibitor epoxomicin, and then the generated epitope-specific CTLs were added. Cytotoxic activity was determined by the ^51^Cr-release assay at the indicated E:T ratios. Values correspond to means (±SD) of percentages of lysis from triplicates. **b** Production of IFN-γ by patient 1’s T cell clones or cloids stimulated with autologous ppCT-expressing tumour cell lines. Anti-ppCT peptide T cells were stimulated for 36 h with IGR-Heu-TAP (anti-ppCT_9–17_, -ppCT_50–59_ and -ppCT_91–100_) or IGR-Heu (anti-ppCT_16–25_) tumour cells, untreated or pretreated with epoxomicin or DCI; then IFN-γ production was measured by ELISA. Data shown are means (±SD) of three T cell clones and T cell cloids from three independent experiments. **c** Involvement of SP in processing of ppCT_9–17_, ppCT_50–59_ and ppCT_91–100_ antigenic peptides. IGR-Heu-TAP tumour cells were incubated with SP inhibitor DCI before addition of anti-ppCT epitope CTLs. Cytotoxicity of anti-ppCT_16–25_ CTLs towards IGR-Heu tumour cells, untreated or pretreated with DCI, was included. **d** Processing of the ppCT_9–17_ epitope involves SPP. The lytic activity of ppCT_9–17_-, ppCT_50–59_- and ppCT_91–100_-specific CTLs against IGR-Heu-TAP, electroporated with siRNA targeting SPP (siRNA SPP) or siRNA control (siRNA Crtl), was examined as in **a**. **e** Processing of ppCT_50–59_- and ppCT_91–100_ epitopes is ERAD dependent. IGR-Heu and IGR-Heu-TAP tumour cells were incubated in the absence or presence of EER1, and then epitope-specific CTLs were added. Values correspond to means (±SD) of percentage of lysis from triplicates. Data shown are from two independent experiments out of three. *p < 0.05; **p < 0.01; ***p < 0.001 (two-tailed Student’s unpaired *t* test). Blue: patient 1, red: patient 13 and green: patient 3. DCI dichloroisocoumarin, EER1 eeyarestatin 1, E:T effector:target

The above results suggested that ppCT_9–17_, ppCT_50–59_ and ppCT_91–100_ epitopes are processed by a TAP-dependent mechanism. Therefore, we examined the involvement of the proteasome/TAP pathway in ppCT peptide processing using the proteasome inhibitor epoxomicin and small interference RNA (siRNA) targeting TAP1. Results indicated that epoxomicin inhibited the cytotoxicity of ppCT_9–17_- ppCT_50–59_- and ppCT_91–100_-specific CTL towards IGR-Heu-TAP tumour cells (Fig. 3a). In contrast, it induced a slight increase in the cytotoxicity of ppCT_16–25_-specific CTLs generated from patient 1. Epoxomicin also inhibited IFN-γ release by ppCT_9–17_-, ppCT_50–59_- and ppCT_91–100_-specific T cell cloids stimulated with IGR-Heu-TAP tumour cells, while it had only a marginal effect on IFN-γ release by ppCT_16–25_-specific T cell clones stimulated with IGR-Heu (Fig. 3b). As expected, downregulation of TAP1 in IGR-Heu-TAP tumour cells resulted in an increase in their susceptibility to anti-ppCT_16–25_ CTL-mediated lysis, while it resulted in a decrease in anti-ppCT_9–17_, anti-ppCT_50–59_, anti-ppCT_91–100_ and CTL-mediated killing (Supplementary Figure 4a).

Because the ppCT_9–17_ peptide is part of the ppCT signal sequence^27^, it is presumably dependent on SP. Accordingly, preincubation of IGR-Heu-TAP with the serine protease inhibitor dichloroisocoumarin (DCI)^28^ markedly inhibited cytotoxicity of CD8^+^ T cells generated towards ppCT_9–17_, as well as ppCT_50–59_ and ppCT_91–100_ (Fig. 3c). DCI also inhibited IFN-γ release of all ppCT peptide-specific T cell cloids and clones generated from patient 1 and stimulated with autologous IGR-Heu-TAP or IGR-Heu tumour cells (Fig. 3b). The involvement of SPP in the processing of ppCT_9–17_ was then demonstrated using specific siRNA, which inhibited lysis of CTL generated towards the ppCT_9–17_ epitope (Fig. 3d). These results indicate that cleavage of the ppCT signal peptide by SP is required for further processing of all ppCT epitopes. They also suggest that, after cleavage by SPP, a ppCT_1–17_ signal peptide fragment is released into the cytoplasm to be further processed by the proteasome and that the generated ppCT_9–17_ peptide is then translocated to the ER lumen by TAP. Accordantly, further downregulation of TAP1 in IGR-Heu tumour cells using specific siRNA (Supplementary Figure 4b) induced a decrease in the lytic activity of ppCT_9–17_ epitope-specific CTLs (Supplementary Figure 4c).

We next examined the role of the ERAD pathway in processing of the pCT precursor protein by treating IGR-Heu and IGR-Heu-TAP tumour cells with the eeyarestatin 1 (EER1) inhibitor. Results included in Fig. 3e show that EER1 inhibited target cell killing by anti-ppCT_50–59_ and anti-ppCT_91–100_ CTLs, while it had only a marginal effect on pCT_9–17_- and ppCT_16–25_-specific CTLs. In these experiments, we verified that DCI, epoxomicin, EER and siRNA targeting SPP and TAP1 had no or only a weak effect on tumour cell viability (Supplementary Figure 4d) and cell surface expression of HLA-A2 molecules (Supplementary Figure 4e). These results indicate that the pCT prehormone undergoes retrotranslocation from the ER into the cytosol to be processed by the proteasome/TAP pathway. The generated ppCT_50–59_ and ppCT_91–100_ epitopes, as well as the ppCT_9–17_ epitope, are then loaded into the ER on HLA-A2 molecules to be presented at the target cell surface.

### ppCT triggers CTL responses towards APM-impaired tumours

**Fig. 4:**
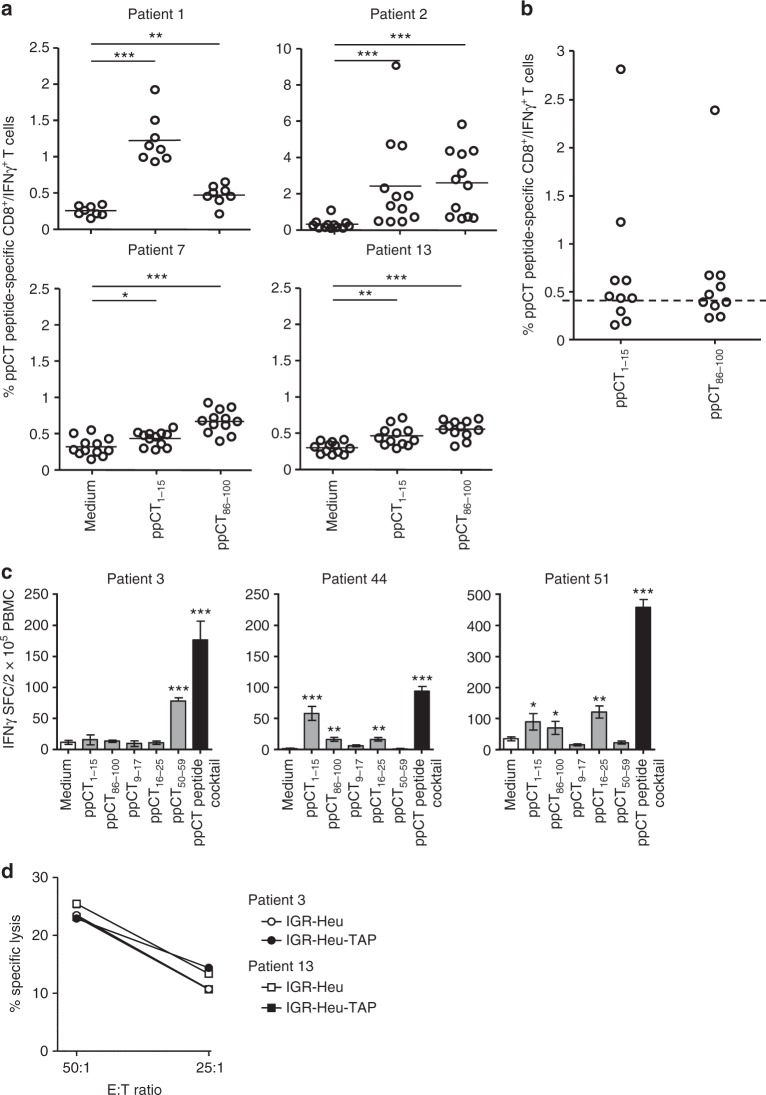
Immunogenicity of ppCT 15-aa-long peptides and a peptide cocktail in patients’ PBMCs. **a** Cytoplasmic expression of IFN-γ in CD8^+^ T cells as determined by intracellular immunofluorescence analysis. NSCLC patients’ PBMCs were stimulated with ppCT_1–15_ or ppCT_86–100_ peptides, then surface-labelled with anti-CD8 mAb and cytoplasmic-labelled with anti-IFN-γ mAb. Percentages of positive cells from 4 patients’ PBMCs are included. *p < 0.05; **p < 0.01; ***p < 0.001 (two-tailed Mann–Whitney *U* test). **b** Mean percentages of IFN-γ-producing CD8^+^ T cells from 10 analysed NSCLC patients’ PBMCs. The horizontal dashed line corresponds to the cutoff value (e.g. mean of the medium condition mean values plus 2 times the SD) above which responses are considered positive. **c** IFN-γ-producing CD8^+^ T cells. PBMCs from NSCLC patients were stimulated with the indicated peptides or the peptide cocktail; then IFN-γ SFC were evaluated by Elispot assay. Values correspond to means (±SD) of SFC from triplicates. Data shown are from three patients. *p < 0.05; **p < 0.01; ***p < 0.001 (two-tail Student’s unpaired *t* test with Welch’s correction). **d** Cytotoxicity of ppCT peptide cocktail-specific CD8^+^ T cells towards IGR-Heu and IGR-Heu-TAP tumour cells. Values correspond to means (±SD) of percentages of lysis from triplicates. Data shown represent experiments from two patients. SFC spot-forming cells, E:T effector:target

Synthetic long peptides constitute a promising vaccine strategy for inducing therapeutic T cell responses in patients with tumours. Therefore, we selected from the ppCT precursor protein two 15-aa-long peptides, ppCT_1–15_ and ppCT_86–100_, each of which included at least three additional peptides predicted to bind to HLA-A2 (ppCT_2–10_, ppCT_4–12_ and ppCT_7–15_ and ppCT_87–95_, ppCT_92–100_ and ppCT_91–99_, respectively; see Supplementary Table 1). These 15-aa-long peptides induced specific IFN-γ-secreting CD8^+^ T cells in PBMCs from 7 out of 10 and 5 out of 10 NSCLC patients, respectively (Fig. 4a, b). We then identified a cocktail of five peptides, including the three ppCT leader sequence-derived peptides (ppCT_1–15_, pCT_9–17_ and ppCT_16–25_), ppCT_50–59_ and ppCT_86–100_, able to induce strong CD8^+^ T cell responses in a majority of patient PBMCs, as monitored by IFN-γ production (Fig. 4c) and killing of IGR-Heu and IGR-Heu-TAP tumour cells (Fig. 4d).

**Fig. 5:**
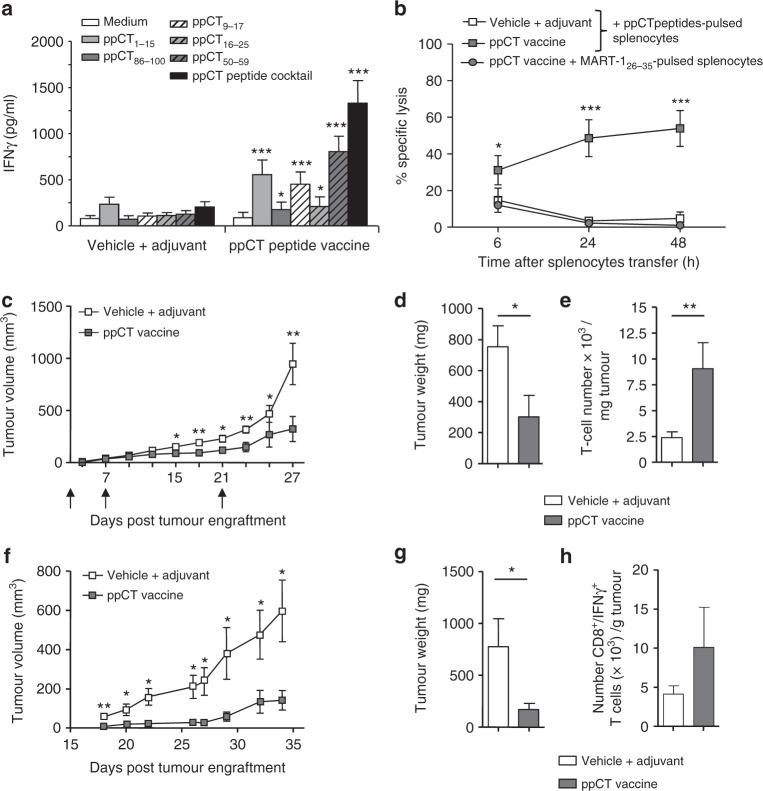
Immunogenicity and antitumour effect of ppCT vaccine. **a** In vivo immunogenicity in HHD-DR3 mice. Mice were immunized four times at 1-week intervals with the ppCT vaccine or vehicle plus adjuvant control. One week after the final immunization, splenocytes were recovered and cultured in medium or restimulated with each peptide or the peptide cocktail; then IFN-γ secretion was measured. Three-to-five mice per group were included. Results are means (±SEM) of three independent experiments (*n* = 12). **b** In vivo cytotoxic activity. Splenocytes were loaded with ppCT peptides and injected i.v. 2 days after the last immunization. Surviving target cell frequencies were detected in blood 6, 24 and 48 h later. Five mice per group were included. Results are means (±SEM) of two independent experiments (*n* = 10). **c** Tumour progression. 10^6^ D122-HHD-ppCT cells were injected s.c. At days 1, 7 and 21 (arrows), mice were vaccinated with ppCT vaccine or vehicle plus adjuvant and tumour volume was measured every third day. **d** Tumour weight. Tumours were recovered at day 27 after engraftment and weighed. **e** Absolute TIL counts. Numbers of TILs per milligram of tumour. Three-to-five mice per group were included. Results in **c**–**e** are given as means (±SEM) of four independent experiments (*n* = 16). **f** IGR-Heu tumour growth in NSG mice. NSG mice were engrafted with IGR-Heu tumour pieces and, at day 10, adoptively transferred with healthy donor PBMCs. At days 11 and 17, mice were vaccinated i.v. with the ppCT vaccine or with vehicle plus adjuvant, and tumour growth was recorded every 2 days. **g** Tumour weight. Tumours were recovered at day 34 after engraftment and weighed. Bars are mean ± SEM. Results are from one experiment out of six. **h** Absolute number of human IFN-γ-producing CD8^+^ T cells. Tumours were recovered, dissociated and human CD45^+^ leukocytes were isolated and restimulated with the ppCT peptide cocktail. Total number of IFN-γ-producing CD3^+^CD8^+^ cells was determined. Five mice per group were included. Results in **f**–**h** are means (±SEM) of one experiment out three (*n* = 16). *p < 0.05; **p < 0.01; ***p < 0.001 (two-tailed Student’s unpaired *t* test)

Next, we tested whether the selected ppCT peptide cocktail could prime ppCT-specific CTLs capable of controlling growth of APM-impaired tumours in vivo. Initial experiments performed in HHD-DR3 transgenic mice immunized with each of the HLA-A2-restricted epitopes, co-administered with the TLR3 agonist polyinosinic–polycytidylic acid (poly(I:C)) adjuvant, showed that ppCT_9–17_ and ppCT_50–59_ induce peptide-specific IFN-γ-producing cells in the spleens of immunized mice (Supplementary Figure 5a). In contrast, dimethyl sulphoxide (DMSO) alone or combined with poly(I:C) had no effect on cytokine production (Supplementary Figure 5b). Moreover, the induced CD8^+^ T cells were able to trigger cytotoxic activity towards IGR-Heu-TAP and/or IGR-Heu tumour cells that was inhibited by anti-MHC-I mAb (Supplementary Figure 5c). Results also indicated that mice immunized with the cocktail of five ppCT peptides administered together with poly(I:C) adjuvant (hereafter named ppCT active immunotherapy or therapeutic ppCT peptide vaccine) developed specific CD8^+^ T cells able to produce IFN-γ when restimulated ex vivo either with specific peptides alone, mainly ppCT_1–15_, ppCT_9–17_ and ppCT_50–59_, or with the peptide cocktail (Fig. 5a). Cytotoxicity of the induced ppCT-specific T cells was also examined in vivo 2 days after the last immunization with the ppCT peptide vaccine following injection of syngeneic splenocytes loaded with the peptide cocktail. Strong killing activity towards specific ppCT peptide-loaded splenocytes, but not towards MART-1_26–35_ peptide-loaded splenocytes used as a control, was seen 24 h after target cell administration, supporting the role of ppCT-specific CTLs in controlling tumour progression (Fig. 5b).

We then examined the antitumour response of the ppCT-based vaccine formulation in HHD-DR3 mice engrafted with the weakly immunogenic D122 clone of Lewis lung carcinoma cell line LL2, transgenic for HLA-A2 (D122-HHD) and transfected with a human ppCT-encoding lentivirus (D122-HHD-ppCT; Supplementary Figure 5d), expressing intermediate levels of HLA-A2 molecules (Supplementary Figure 5e). Results indicated that D122-HHD-ppCT tumours grew much more slowly in vaccinated than in non-vaccinated (DMSO plus poly (I:C)) mice (Fig. 5c, d). Moreover, control of tumour growth in vaccinated mice was correlated with much stronger tumour infiltration with T lymphocytes than in non-vaccinated mice (Fig. 5e). To further assess the capacity of ppCT-based immunotherapy to control progression of APM-impaired human tumours, we developed a preclinical model of NSG mice engrafted with the human ppCT^+^ IGR-Heu tumour, expressing low levels of HLA-A2 (Supplementary Figure 5e), and then adoptively transferred with human healthy donor PBMCs previously tested in vitro for their capacity to induce a T cell response towards ppCT epitopes. Data showed a delay in tumour growth in mice treated with ppCT peptide vaccine compared to mice treated with DMSO plus poly (I:C) control (Fig. 5f, g). Moreover, an increase in the absolute number of human CD8^+^ T cells producing IFN-γ when restimulated ex vivo with the peptide cocktail was observed in tumour-infiltrating lymphocytes (TILs) from vaccinated mice as compared to non-vaccinated mice (Fig. 5h). Overall, these data indicate that ppCT peptide-based active immunotherapy triggers an efficient antitumour T cell response towards APM-impaired tumours and that it corresponds to a promising strategy for treatment of ppCT-expressing tumours that have escaped conventional immunotherapies.

## Discussion

In the present study, we report on T lymphocytes specific to the ppCT tumour antigen and on CD8-T cell-defined ppCT-derived epitopes processed by proteasome/TAP-independent and -dependent mechanisms. We also report here that human lung tumours frequently express the ppCT precursor protein and that most of them display defects in TAP1 and/or TAP2 expression. Deficiencies in TAP subunits have been described in several human cancer types, including cervical^29^, head and neck^30^, melanoma and gastric^31–33^ cancers, and were associated with tumour escape from CD8 T cell immunity. Thus CTLs specific for such antigen-processing mutants and their target antigens have been identified for use in preventing cancer immune evasion and designing more effective anticancer therapeutic strategies. Interestingly, most of these CTLs were found to recognize epitopes derived from signal peptides of precursor proteins that are independent of cytosolic processing and transport pathways^34–36^. Among rare known epitopes belonging to this class of antigens are the human HLA-A2-restricted melanoma-associated tyrosinase 1–9 epitope^37^, the ppCT_16–25_ epitope^22,23^ and the murine H2-D^b^-restricted C-terminal peptide epitope of TRH4 ceramide synthase^38^. While the human ppCT_16–25_ epitope is derived from the C-terminal region of the preprotein signal peptide and is processed by the SP and SPP pathway^22^, the murine TRH4 epitope is generated by SPP independently of the SP^38^.

After cleavage by SPP, signal peptide fragments can be released either into the ER, where they follow TAP-independent processing^39^, i.e. the ppCT_16–25_ epitope^22^, or into the cytoplasm, to be processed by the proteasome/TAP pathway, i.e. signal peptide-derived epitopes presented by HLA-E molecules^40^. Here we provide evidence that the ppCT signal peptide generates an additional HLA-A2-restricted epitope, ppCT_9–17_, the processing of which is dependent not only on SP (required to release the leader sequence from the precursor protein) but also on SPP and presumably also TAP. These results are in agreement with the observation that signal sequences that have been cleaved by SP can be further processed by SPP and then by cytosolic proteases^39^. In *Escherichia coli*, cleaved signal sequences are processed by membrane-bound protease IV and further degraded by cytosolic oligopeptidase^41^. In eukaryotic cells, processing of signal peptide fragments can also occur in the cytosol^42^ and then bind to MHC-I molecules in the ER lumen^43–45^. Likewise, the ppCT signal peptide is cleaved by SPP to generate the ppCT_16–25_ epitope within the ER, and a ppCT_1–17_ peptide, which is released into the cytosol, to be further processed by the proteasome to generate the ppCT_9–17_ epitope, which is then likely translocated to the ER lumen by TAP. Therefore, fragments from leader sequences with a type II signal anchor^39^, like the ppCT signal peptide^22,46^, are released into the ER lumen and presented to CTLs by MHC-I molecules in a TAP-independent manner (such as the ppCT_16–25_ epitope) or into the cytosol where they are further processed by the proteasome (like the ppCT_1–17_ peptide). Thus it makes sense that the resulting fragments (such as the ppCT_9–17_ epitope) would be transported by TAP into the ER lumen, where they bind to the MHC-I molecules and then conveyed to the cell surface to be recognized by CTLs. Generation of both ppCT_16–25_ and ppCT_9–17_ epitopes by SPP supports the hypothesis that the cleavage site of this aspartic protease in the centre of the h-region of the ppCT signal peptide is approximate and may lead to different peptide fragments. The ppCT_9–17_ epitope is most likely naturally processed by tumour cells, since the generated CTL were able to lyse ppCT-expressing target cells.

Apart from its leader sequence, ppCT can also generate HLA-A2-restricted CTL epitopes from the N-terminal region of preprotein (ppCT_50–59_) or the hormone itself (ppCT_91–100_). Their processing also requires cleavage of the signal peptide by SP and the ERAD pathway, which involves substrate retrotranslocation from the ER lumen into the cytosol for degradation by the proteasome/TAP pathway. Indeed, after cleavage of its signal peptide, with type II orientation, i.e. spanning the ER membrane with the n-region exposed towards the cytosol and the c-region facing the ER lumen^22,46^, pCT is released into the ER, where it is subsequently transferred across the membrane, via ERAD^47–49^, to enter the cytosol and then follow proteasome/TAP-dependent processing. Accordingly, processing of ppCT_50–59_ and ppCT_91–100_ is inhibited not only by DCI but also by EER1 and epoxomicin, which inhibit SP, ERAD and proteasome, respectively. These results support the conclusion that release of the signal peptide is a prerequisite for further processing of the preprotein, which can occur either in the ER by the SP/SPP pathway (ppCT_16–25_) or in the cytosol after cleavage by SPP (ppCT_9–17_) or retranslocation of the pCT from the ER lumen via the ERAD pathway (ppCT_50–59_ and ppCT_91–100_).

Identification of TAA in solid tumours, in particular, melanoma, led to development of therapeutic peptide-based cancer vaccines, the aim of which is to activate or reactivate tumour-specific CTLs. Therapeutic peptide vaccinations with TAA, including differentiation antigens, overexpressed antigens, cancer/testis antigens and viral antigens, are currently under investigation, and results are promising^50^. Transfer of in vitro-activated TILs^8,9^ and blockade of inhibitory receptors such as CTLA-4 and PD-1 have led to impressive results, with survival benefits in many cancers, including NSCLC^3–6^. Success of cancer immunotherapies, including cancer vaccines, has been associated with (re)activation of T lymphocytes specific to neoantigens arising from DNA mutations in tumour cells^8,9,12–17^. Unfortunately, only a subset of patients responds to these therapies, indicating that tumours are able to use additional resistance mechanisms to escape immunotherapy-induced antitumour T cell responses. Among these mechanisms, alterations in tumour APM play an important role. In this context, it has been shown that defects in the HLA-class I APM may occur in malignant cells after active immunotherapy^51^ and PD-1 blockade immunotherapy^20^. Some of these defects include an irreversible tapasin mutation associated with loss of *HLA* genes, a truncating mutation in the gene encoding β2m and loss of TAP subunits^52–57^. Thus self-antigens belonging to TEIPP are particularly attractive because they emerge on cancer cells with defects in APM and thus enable overcoming tumour escape from CD8 T cell immunity.

The ppCT-based immunotherapy that we have developed here includes at least one TEIPP (ppCT_16–25_), two additional peptides derived from the ppCT signal peptide (ppCT_9–17_ and ppCT_1–15_) and two peptides derived from the pCT (ppCT_50–59_ and ppCT_86–100_). Inclusion of proteasome/TAP-independent and -dependent HLA-A2-restricted epitopes would enable targeting cancer cells with either an impaired or functional proteasome/TAP pathway and thus overcoming tumour evasion from CTL attack. Moreover, the ppCT-based therapeutic vaccine includes two 15-aa-long peptides (ppCT_1–15_ and ppCT_86–100_) that would permit activating CD8^+^ and possibly CD4^+^ ppCT-specific T lymphocytes. This active cancer immunotherapy, delivered with the TLR3-ligand poly(I:C) as adjuvant, resulted in pronounced progression delay of lung tumours displaying impaired APM established in HLA-A2 transgenic mice or NSG mice that were adoptively transferred with healthy donor PBMCs. Tumour growth control was associated with induction of ppCT-specific CD8^+^ T cells, including ppCT_16–25_ TEIPP-specific T cells. TEIPP-specific T lymphocytes were also found to be activated by therapeutic vaccination with synthetic long peptides composing the minimal CD8 T cell epitope^21^. These results suggest that TAP-proficient host antigen-presenting cells were able to process these long peptides and to cross-present TEIPP in MHC-I molecules, in the context of a fully competent peptide repertoire.

Overall, our findings provide in vitro and in vivo proof of concept of a ppCT-based therapeutic cancer vaccine and support the conclusion that signal sequence-derived peptides and their carrier proteins are attractive candidates for specific active cancer immunotherapy. They also provide a rational design of combinatorial cancer immunotherapy harnessing a ppCT-based peptide vaccine, together with checkpoint inhibitors, in particular anti-PD-1 and anti-PD-L1 mAbs, to treat patients suffering from NSCLC, MTC and NET and to prevent outgrowth of immune-escaped cancer cells.

## Methods

### Healthy volunteers and patients

Healthy donor blood samples were collected from the French blood bank (Etablissement Français du Sang (EFS); agreement number No. 12/EFS/079), and patient samples were collected from Gustave Roussy. All patients were suffering from advanced and inoperable NSCLC stage IIIB/IV. Blood samples were drawn from patients after induction chemotherapy. Immune monitoring in the blood of patients was approved by the Kremlin-Bicêtre Hospital Ethics Committee (no. 110–08; ID RCB: 2008-A01171–54), and Declaration of Helsinki protocols were followed. Healthy donors and patients provided their written informed consent prior to inclusion in this study.

### Real-time qRT-PCR and IHC staining

Fresh NSCLC tumours and normal lung tissues of 32 patients were obtained from the Centre chirurgical Marie Lannelongue and the Institut Mutualiste Montsouris. RNA were immediately extracted with TRIzol reagent (Invitrogen), reverse-transcribed and then subjected to qRT-PCR^22^. RNAs from a pool of five human healthy thyroid tissues^58^ were included. PCR probes for *TAP1*, *TAP2* and *CALCA* genes were designed by Applied Biosystems (*TAP1*: Hs00184465_m1; *TAP2*: Hs00241066_m1; *CALCA*: Hs00266142_m1) and used according to the manufacturer’s recommendations.

FFPE primary tumour samples were obtained from patients diagnosed with early-stage NSCLC^59^. A total of 215 tumour samples, including 31% ADC, 16% squamous cell carcinomas, 27% NET, 22% undifferentiated tumour and 4% other subtypes were tested by IHC for CT expression using anti-CT (Dako, ref. A0576, dilution 1/2000) Ab. From this cohort, a total of 135 FFPE tumour samples were tested by IHC for TAP protein expression using anti-TAP2 Ab (dilution 1/20) produced in one of our laboratories^60^. Briefly, 4-μm-thick whole sections from FFPE lung cancer specimens were mounted on poly-l-lysine-coated slides, deparaffinized and rehydrated through graded alcohol to water. Antigen retrieval was performed in a citrate buffer (pH = 6) for 30 min at 98 °C. Endogenous peroxidase activity was inhibited with 3% hydrogen peroxidase (Sigma Aldrich) for 10 min, and non-specific proteins were blocked for 15 min. The primary Ab was incubated for 1 h at room temperature (RT). Immunostaining was visualized using goat anti-rabbit horseradish peroxidase (HRP) 1:100 (Powervision, Leica, ref. PV6119) for 20 min at RT and then adding 3,3'diaminobenzidine substrate (Powervision, Leica). Slides were counterstained with Mayer’s haematoxylin (VWR).

### Antibodies and immunofluorescence analyses

Anti-human CD8-APC (ref. 130–091–076, dilution 1/100) and anti-IFN-γ-PE (ref. 130–113–493, dilution 1/50) mAbs were purchased from Miltenyi Biotech. Anti-human CD45-PE-Cy7 (ref. 25–0459–42, dilution 1/200), anti-CD3-FITC (ref. 11–0038–42, dilution 1/200), anti-CD4-PE (ref. 12–0048–42, dilution 1/200), anti-IFN-γ-APC (ref. 17–7319–82, dilution 1/100) and anti-mouse anti-CD8-alexafluor 700 (ref. 56–0081–82, dilution 1/200) mAbs were purchased from Thermofisher Scientific. Anti-human CD8-Pacific-Blue (ref. 301026, dilution 1/200) and anti-mouse CD3-APC-Cy7 (ref. 100222, dilution 1/200) mAbs were provided by Biolegend. Anti-HLA-A2 (BB7.2 and MA2.1, dilutions 1/1000 and 1/400, respectively) and anti-MHC-I (W6/32, dilution 1/100) mAbs were purified from ascitic fluids in one of our laboratories.

Expression of surface molecules was performed by immunofluorescence analyses using specific mAb. For cytoplasmic IFN-γ expression, human PBMCs were stimulated for 6 h at 37 °C with 2.5 µM of each peptide in the presence of 10 µg/ml Brefeldin A (Sigma, ref. B6542). After anti-CD8 staining, cells were fixed with phosphate-buffered saline (PBS) containing 2% formaldehyde; their membrane was permeabilized using PBS supplemented with 0.5% bovine serum albumin and 0.2% saponin and then stained with anti-IFN-γ mAb. Samples were analysed using an Accuri C6 cytometer or Fortessa cell analyser (BD Biosciences), and data were processed by the Cflow software (BD Biosciences) or FlowJo software (Tree Star Inc).

### Peptide prediction, HLA-A*0201 binding and stability assays

The ppCT sequence was scanned for HLA-A*0201-binding peptides using the prediction software SYFPEITHI (www.syfpeithi.de). Three 9–10-aa-long peptides, ppCT_9–17_ (FLALSILVL), ppCT_50–59_ (LLAALVQDYL) and ppCT_91–100_ (CMLGTYTQDF), were selected from a ppCT signal peptide (ppCT_9–17_) and pCT (ppCT_50–59_ and ppCT_91–100_) precursor protein. Two 15-aa-long peptides, ppCT_1–15_ (MGFQKFSPFLALSIL) and ppCT_86–100_ (GNLSTCMLGTYTQDF), were also selected because they include additional predicted HLA-A2-restricted peptides. Peptides were synthesized by Genescut or Proteogenix at a purity of >75%. Lyophilized peptides were dissolved in DMSO at a concentration of 10–100 mM and stored at −80 °C.

To determine whether the candidate peptides can bind to HLA-A*0201, upregulation of peptide-induced HLA-A2 molecule expression on T2 cells (ATCC® CRL-1992™) was examined. Briefly, 3 × 10^5^ T2 cells were incubated with 100 µM of the synthesized peptides and 100 ng/ml of human β2m (hβ2m, Sigma) in serum-free RPMI 1640 medium for 16 h at 37 °C. T2 cells incubated with hβ2m alone served as a negative control. Expression of HLA-A*0201 on T2 cells was then examined by staining with BB7.2 mAb, followed by fluorescein isothiocyanate (FITC)-labelled goat-anti-mouse IgG (Biolegend, ref. 405305, dilution 1/100) secondary Ab. The fluorescence index (FI) was calculated as follows: FI = (mean fluorescence intensity (MFI) with the given peptide − MFI without peptide)/MFI without peptide^61^. The HLA-A2.1-restricted Melan-A/MART-1_26–35_ peptide served as positive control^62^.

To test peptide/HLA-A*0201 complex stability, T2 cells were incubated at 37 °C overnight with 100 μM of each peptide in serum-free RPMI 1640 medium supplemented with 100 ng/ml of hβ2m and washed to remove free peptides. They were then incubated with 10 μg/ml of brefeldin A for 1 h to block newly synthesized HLA-A*0201 molecules from being expressed on the cell surface, washed and incubated at 37 °C for 30 min, 1, 2, 4, 6, 8 or 12 h. Subsequently, cells were stained with BB7.2 mAb and FITC-labelled goat anti-mouse IgG. For each peptide, the equation of linear trend line was determined and the half-life (DC_50_) of the HLA-A2-peptide complex was calculated.

### In vitro T cell stimulation with synthetic peptides

For induction of peptide-specific CTLs, PBMCs were isolated using Ficoll-Paque and incubated for 1 h at 37 °C with 20 µM of each peptide in RPMI medium supplemented with 1% human AB serum (SAB). Peptide-pulsed PBMCs were washed and plated at 2 × 10^5^ cells/0.2 ml in U-bottom 96-microwell plates, in RPMI medium with 10% SAB, 1 % sodium pyruvate, penicillin (100 U/ml), streptomycin (10 μg/ml), IL-2 (20 U/ml, Miltenyi Biotech, ref. 130–097–745), IL-4 (10 ng/ml, Miltenyi Biotech, ref. 130–093–922) and IL-7 (10 ng/ml, Miltenyi Biotech, ref. 130–095–363). At day 7, cells were restimulated with the same medium supplemented with 20 µM peptide, and 1 week later, each microplate column was harvested and T cell functional activities were analysed. T cell clones and T cell cloids specific to ppCT peptides were generated from patient 1 by limiting dilution^63–65^.

### Functional assays

IFN-γ secretion was measured using Ready-Set-Go enzyme-linked immunosorbent assay (ELISA) according to the manufacturer’s recommendation (eBioscience, ref. 88–7316). Briefly, 96-well plates were coated overnight with anti-mouse or anti-human IFN-γ mAb (capture Ab) at 4 °C. Then plates were washed with PBS–Tween 20 and saturated for 1 h. Supernatants were diluted to 1/10, transferred to plates and incubated for 2 h at RT. After washing, plates were further incubated with biotinylated anti-mouse IFN-γ mAb (detection Ab) for 1 h at RT. Plates were washed, incubated for 30 min at RT with avidin-HRP and then developed by the addition of substrate solution (TMB), followed by 10–15 min of incubation at RT in the dark. The enzymatic colour development was stopped by addition of sulphuric acid solution (2 N), and the optical densities of each well were read at 450 and 570 nm using a microplate reader (Opsys MR, Dynex Technologies). The values of 570 nm were subtracted from those of 450 nm and data were analysed. IFN-γ concentrations in supernatants were determined from the standard curve and expressed as pg/ml.

The peptide-specific T cell response induced after in vitro stimulation of patient PBMCs with ppCT-derived peptides was examined using the Elispot assay according to the manufacturer’s recommendation (Diaclone, Ozyme, ref. 856.051). Briefly, 96-well plates containing nitrocellulose filters (Multiscreen; Millipore) were coated overnight with anti-IFN-γ mAbs (capture Ab) at 4 °C. The plates were washed with PBS–Tween 20 and saturated for 2 h with RPMI medium, 10% SAB. One week after the last stimulation, PBMCs were cultured in triplicate at 2 × 10^5^ cells/well and restimulated with peptides at 20 µM. For positive control, PBMCs were stimulated with 1 ng/ml phorbol myristate acetate (PMA) and 500 ng/ml ionomycin. After 16 h, plates were washed with PBS–Tween 20 and incubated for 10 min at 4 °C. Plates were then washed with PBS–Tween 20 and incubated for 1 h 30 min with biotinylated anti-mouse IFN-γ and 1 h with alkaline-phosphatase-conjugated streptavidin. Spots were developed by adding phosphatase substrates, 5-bromo-4,3-indolyl phosphate;nitroblue tetrazolium (BCIP/NBT) and spot-forming cells (SFC) were counted using the CTL Immunospot system (Cellular Technology Ltd). Cells stimulated with PMA (1 ng/ml, Sigma, ref. P1582) and ionomycin (500 ng/ml, Sigma, ref. I0634) were included as positive controls. The negative control consisted of cells cultured in medium alone. A response was considered positive if the number of spots in the well containing cells stimulated with peptides was at least 2-fold higher than the number of spots in the well containing cells without peptide, with a cutoff of 10 SFC.

In vitro T cell cytotoxic activity was measured by a conventional 4 h chromium (^51^Cr) release assay^66^. CD8^+^ T cells, isolated by magnetic beads using the CD8^+^ T Cell Isolation Kit (Miltenyi Biotech, ref. 130–096–495), and T cell clones and cloids were used as effector cells. For proteasome, SP and ERAD inhibition, 10^6^ tumour cells were resuspended in media in the presence of specific inhibitors. Briefly, tumour cells were incubated for 2 h at 37 °C with epoxomicin (10 µM, Merck Millipore, ref. 324800), DCI (250 μM, Merck Millipore, ref. 287815) or EER1 (10 μM, Merck Millipore, ref. 324521)^67,68^, washed, resuspended in acid buffer for 30 s, washed and then incubated for an additional 3 h in the presence of each inhibitor. None of the inhibitors were toxic at the given concentrations, as tested by the Annexin V Apoptosis Detection Kit I (BD Pharmingen, ref. 556547). The parental tumour cell line IGR-Heu, TAP1- and TAP2-transfected cell line IGR-Heu-TAP, generated from patient 1 (Heu)^23^, the lymphoblastoid B cell line Heu-EBV (established in one of our laboratories) and human erythroleukaemia cell line K562 (ATCC® CCL-243™) were used as targets. All the cell lines are mycoplasma-free and were regularly tested for mycoplasma contamination. We regularly authenticate IGR-Heu and IGR-Heu-TAP cell lines by testing recognition by autologous CTL clones, such as ppCT-specific clones, and HLA-A2 and TAP expression. Gene silencing of SPP (HM13, sense 5′-gac aug ccu gaa aca auc a dtdt-3′, and antisense 5′-uga uug uuu cagg caug uc dtdg-3′, Ambion, ref. 202377) and TAP1 (HS_TAP1_2_HP, sense 5′-gcc gau acc uuc acu cga a dtdt-3′ and antisense 5′-uuc gag uga agg uau cgg c dtdg-3′, Qiagen, ref. SI00012418) expression in IGR-Heu target cells was performed using sequence-specific siRNA^22,23^.

### Mice, in vivo experiments and immunization protocol

HHD-DR3 (mice humanized for both HLA-A2 and HLA-DR3 and deleted for both H-2 class 1 and 2 molecules (β2m−/− H-2Db−/− IAβ−/− IAα−/− IEβ−/−) and NSG mice were bred and maintained at the animal facility of Gustave Roussy. Animal experiments were performed in accordance with guidelines established by the institutional animal committee (CEEA no. 26: 2012–146 and 2017–081–12717). Eight-to-12-week-old male and female HHD-DR3 transgenic mice, randomized to obtain homogeneous groups in term of gender and age, were immunized subcutaneously (s.c.) with equimolar amounts of peptides (100 μM) emulsified in poly (I:C) adjuvant (Invivogen, ref. vac-pic). One week after the last immunization, cell suspensions from the spleens were cultured for 3 days in RPMI medium supplemented with 20 µM of peptides and 20 U/ml of IL-2, and then IFN-γ secretion was tested by ELISA (ebioscience, ref. 88–7314).

In vivo cytotoxic activity was measured using the VITAL assay^69,70^. Briefly, HHD-DR3 splenocytes were incubated for 2 h either in the presence of DMSO alone, with 10 µM MART-1_26–35_ negative control or with 0.1 or 10 µM ppCT peptide cocktail, and then labelled with 0.5 or 5 µM CellTrace™ Far Red (Molecular Probes, ref. C34564) for non-specifically-loaded (MART-1_26–35_) and unloaded (DMSO) targets, respectively, or with 0.1 or 1 µM carboxyfluorescein succinimidyl ester (CFSE; Molecular Probes, ref. C34554) for 10 and 0.1 µM of ppCT-specific loaded targets, respectively. Labelled cells were mixed at equal ratios and 3 × 10^6^ cells of each population were injected intravenously (i.v.) at 6, 24 and 48 h after target cell injection and blood was collected for flow cytometric analysis. The percentage of specific killing was calculated with the following formula: 100 − [100 × (% CFSE^+^ cells in mice vaccinated/% Far red^+^ cells in vaccinated mice)/(mean of % CFSE^+^ cells in non-vaccinated mice/mean of % Far red^+^ cells in non-vaccinated mice)].

For the antitumour response, HHD-DR3 mice were inoculated s.c. with 1 × 10^6^ of Lewis lung carcinoma D122-HHD-ppCT cells (Lewis lung carcinoma transgenic for HLA-A2, a gift from L Eisenbach, Weizmann Institute, Israel and infected with a lentivirus encoding ppCT in one of our laboratories) and then vaccinated s.c. at days 1, 7 and 21 after tumour engraftment. Tumour volume was measured using a caliper twice a week and estimated using the following formula: length × width × thickness (mm^3^). At day 27, tumours were surgically removed, weighed and dissociated by enzymatic digestion according to the manufacturer’s recommendations (Tumour Dissociation Kit, Miltenyi Biotech, ref. 130–096–730). T lymphocytes from each tumour were then positively selected using anti-CD90.2 mAb-coated Dynabeads according to the standard immunoselection protocol (Dynal, Invitrogen, ref. 11465D). Single-cell suspensions were counted, the percentage of CD3^+^/CD8^+^ lymphocytes was determined by flow cytometry and the number of TILs per milligram of tumour was then calculated.

An immunodeficient mouse model that can be used to analyse the human CTL response to ppCT-based active immunotherapy was developed and adapted from refs. ^71,72^ in accordance with guidelines established by the institutional animal committee (CEEA no. 26: 2012–147 and 2017–081–12717). With this aim, 6–10-week-old male and female NSG mice, randomized to obtain homogeneous groups in term of gender and age, were engrafted s.c. with the patient 1 tumour (Heu-n-IR), which was generated by implantation of the IGR-Heu tumour cell line previously transfected with the intercellular adhesion molecule-1 adhesion molecule and CCL5 (Rantes) chemokine in one of our laboratories, and maintained in nude mice^73^. When tumours were palpable, about 10 days later, mice were injected i.v. with 2 × 10^7^ healthy donor PBMCs. Before transfer into mice, healthy donor PBMCs were tested in vitro for their capacity to induce a CD8 T cell response towards ppCT epitopes. At days 1 and 7 after PBMC engraftment, mice were vaccinated i.v with the ppCT peptide vaccine, and tumour volume was measured every 2 days as described above. Tumours were recovered at day 28 after engraftment, weighed and dissociated as described above. Human CD45^+^ leukocytes were then selected by magnetic beads (Miltenyi Biotech, ref. 130–045–801), restimulated for 6 h with allogeneic HLA-A2^+^ mature dendritic cell pulsed with the ppCT peptide cocktail, and the number of human CD3^+^/CD8^+^ cells secreting IFN-γ per gram of tumour was determined by flow cytometry. In this study, approximately 250 mice were used and divided in groups of 5–10 mice per group. Animals were randomized to obtain homogeneous groups in term of gender and age.

### Statistical analyses

The sample size for in vitro and in vivo experiments were determined by calculation of the statistical power. GraphPad Prism5 software (GraphPad Software, Inc., San Diego, CA, USA) was used for graphic representation and statistical analysis. For in vitro experiments, a two-tailed Mann–Whitney *U* test was applied to statistically analyse the efficiency of ppCT peptides at generating CD8^+^/IFN-γ^+^ T lymphocytes after stimulation of NSCLC patient PBMCs. A two-tailed Student’s unpaired *t* test with Welch’s correction (we do not assume equal variances) was applied to analyse the specific precursor number after peptide stimulation of NSCLC patient PBMCs. A two-tailed Student’s unpaired *t* test was applied to determine peptides processing. Mean ± error bars (standard deviation, SD) was shown. For in vivo experiments, a two-tailed Student’s paired *t* test was used to statistically analyse the immunogenicity of the ppCT peptide vaccine. A two-tailed Student’s unpaired *t* test was used for statistical analysis of the antitumour effect of the ppCT peptide vaccine. Mean ± error bars (standard error of the mean, SEM) is shown.

## Electronic supplementary material

Peer Review File

Supplementary Information

## Data Availability

A reporting summary for this article is available as Supplementary Information file. The authors state that all data generated during this study are included in the article and its supplementary information file and are available from the corresponding author upon reasonable request.
