# Supplementary material for: Human preprocalcitonin self-antigen generates TAP-dependent and -independent epitopes triggering optimised T-cell responses toward immune-escaped tumours
Source: Nat Commun. 2018 Nov 30;9:5097. doi: 10.1038/s41467-018-07603-1 (PMC6269466; doi:10.1038/s41467-018-07603-1)
Supplement: Supplementary file 2 — Supplementary Information [file 41467_2018_7603_MOESM2_ESM.pdf]

Parts of this Peer Review File have been redacted as indicated to maintain the confidentiality of unpublished data.

Reviewers' Comments:

Reviewer #1:
Remarks to the Author:
The paper by Durgeau et al describes interesting data on T cell immunity against lung carcinomas. The authors previously identified a CD8 T cell epitope from the leader sequence of the preprocalcitonin protein and showed that this HLA-A2 binding peptide is presented by TAP-negative lung carcinomas. In the current manuscript they reveal several other T cell epitopes on this protein and demonstrate their dependence on TAP, signal peptidase, signal peptide peptidase and ERAD. Moreover, immunogenicity of these several peptides is tested in lung cancer and healthy PBMC, showing higher CD8 T cell responses in the cancer patients. Finally, mouse models (immunocompetent HLA-A2 transgene and NSG mice) demonstrate the in vivo efficacy of these CD8 T cell reactivity against tumor growth. This paper encompasses a significant amount of work, is coherent, well written and of major interest due to the TAP-dependent and TAP-independent character of the involved tumor antigens. Immunotherapy will result in a selective pressure towards immune escaped tumors with defects in the antigen processing machinery and these novel type of TAP-independent neoantigens might constitute a valid approach to counteract this.

Major issue:
1. In several figures where immunogenicity of the ppCT peptides are portrayed (fig 1, fig 4 and suppl fig 1) the frequencies of IFNg positive CD8 T cells in the cultures are depicted as circles. Do these circles represent repetitive culture wells? Are means depicted (and not median frequencies, as suggested in suppl fig 1)? In figure 1b and 1d the summary of 15 patients are shown, however the medium values are not coupled to the peptide-stimulated values. Therefore, these graphs are not very insightful. It would be much better if T cell responses are scored for each patient on the basis of a biological relevant cut-off value (e.g. mean of medium value plus 2 times the SD). Each response that reaches this threshold might be enumerated. Clearly, a lower percentage of responding patients will be observed, but this is a more standardized way to score immunogenicity and spontaneous anti-tumor CD8 T cell responses are usually low in cancer patients. The data from PBMC of healthy donors (suppl fig 1) should be included in the paper itself to directly compare these in figure 1 with those of cancer patients.

Minor issues:
2. Introduction: the Trh4 derived TEIPP epitope in the mouse is not derived from the signal peptide, but from the C-terminus of the protein (see reference 36).
3. Table I: please clarify in the legend of the table how relative expression was calculated. Were delta Ct values of housekeeping genes normalized to healthy thyroid tissue (CALCA) or normal autologous lung (TAPs)? Also, spell out the abbreviations of disease subtypes. The indicated sample numbers are irrelevant for this table.
4. Table III: How are Fluorescence Index calculated? What does it mean when these values are lower than 1? This suggests lower HLA-A2 expression than control T2 cells.
5. Suppl fig 3: Explain in legend what HHD-DR3 mice are.
6. Fig 5: did vehicle vaccinated mice received DMSO with poly I:C? How many mice were used in fig 5a and how many times were the tumor outgrowth experiments performed?

Reviewer #2:
Remarks to the Author:
The work of Durgeau et al. revolves around an exciting topic which is the recognition of tumors that in principle may escape immunity by down-regulation of antigen presentation machinery (APM) components, such as TAP. In the last couple of years they have published a series of papers in which they studied the immune response to human preprocalcitonin (CT), a self-antigen that was spontaneously recognized by CTL from a lung cancer patient. These CTL preferentially recognized tumor cells with a low TAP expression. The current manuscript presents their data on

their study of the response to this CT self-antigen in many patients as well as provides the first evidence that representative peptides may be used as immunotherapeutic vaccine to control tumor growth.

Major remarks

1. In Tables 1 and 2 they present the data on CT and TAP RNA expression and CT protein expression. At the RNA level 55% (8/14) of the ADC tumor overexpress CT, at the protein level this is only 20%. In SCC 3/6 show overexpression at the RNA level, this is 0% at the protein level. This discrepancy is not explained nor the consequence for their statement that 75% of the lung tumors expressed low levels of TAP1 and TAP2 mRNA. At this point one rather would like to see protein data on TAP, so that a better estimation of the real percentage of tumors with TAP defects can be made.

2. In figure 1 the responses of IFNgamma producing CD8+ T cells from lung cancer patients as measured by flow cytometry is shown. The data presentation and interpretation is unclear and the reader is left with several questions: a) What is the definition of a positive reaction; b) while the M&M section state that per peptide tested one column of a 96-well plate is tested (8 wells) the graphs clearly show more data points for some peptides and less data points for others, suggesting that multiple tests have been combined whereas in other conditions have been left out; c) the PBMC were stimulated for 7 days and then restimulated for the flowcytometric analysis, it is common to compare in such cultures the medium (background production control) and the peptide stimulated cells from the same culture. Currently, it is hard to tell if the IFNg positive CD8 T cells all responded to the peptide during the test. These data should be presented by the authors, furthermore, the authors should provide one flow cytometry example of such a test in the supplementary data.

3. Figure 1b shows the accumulated data of 15 patients. The authors should define what a positive response is and then present a bar graph of the number of responding patients to each peptide. Now their conclusion (page 8, lines 171-173) is hard to follow.

4. To show that the CD8+ T cells responsive to the ppCT peptides can recognize naturally processed peptides presented at the surface of tumor cells, the data in figure 2 is presented. Isolated CD8+ T cells from 3 patients are used. Overall there is only a low level of cytotoxicity shown, generally lower than 20% at the highest E:T ratio. I would consider this suggestive but certainly not definitive proof. The authors should use a more sensitive read-out, such as IFNg production by the T cells upon recognition of the tumor cells. Preferably, the authors should isolate T cell clones from their cultures to make this point.

5. To show the effect of TAP expression, figure 2 also shows the killing of tumor cells that overexpress TAP. Again, while the data is suggestive, the differences in killing between TAP+, wild type and control cells are marginal. T-cell clones to the identified peptides are required to provide final proof.

6. In figure 4, the immunogenicity of peptides is tested using patient's PBMC. Several questions come up: a) Two longer peptides ppCT1-15 and ppCT86-100 are used. As the authors defined an epitope as ppCT9-17, it is not clear to me why they chose to make a long peptide missing the last two amino acids of this epitope. This should be explained, or rather a ppCT1-17 peptide should be used; b) page 11, line 239 states "from most NSCLC patients" but only five are shown; c) a discrepancy exists between the data shown for patient 3 in fig 4a (positive response to ppCT1-15) and fig4b (no response to this peptide; d) fig 4b, patient 20 shows a response to the ppCT peptide cocktail but not to the individual peptides, this is strange as at least one peptide of the cocktail should be positive.

7. In figure 5 data is shown on their experiments in two different mouse models. These experiments can be considered essential. In the tumor control experiments (figures 5c and 5f)

they show vaccinated mice or mice injected with vehicle only. It is important to realize that vaccination comprises the injection of peptide with poly I:C, whereas the vehicle control is DMSO only. The proper control to show that the tumor control is mediated by peptide-induced T cells and not by the adjuvant is to also inject the control group with the adjuvant (poly I:C). In figure 5c they use the Lewis cancer cell line LL2 as a target tumor. Previously it has been published (Shime et al. PNAS 2012, p2066) that poly I:C injection controls tumor outgrowth of Lewis cancer cells via the repolarization of tumor associated macrophages. This effectively means that currently there is no formal proof that the vaccine-activated T cells control tumor outgrowth in this experiment. Also the increase in CD8 T cells in the tumor can be an indirect effect of changes in TAMs.

8. With respect to the data presented in figure 5f/g where NSG mice are injected with human PBMC and the IGR-heu tumor, the same arguments apply. Furthermore, as the authors state that the injected PBMC after vaccination control tumor outgrowth, they should also show that vaccination leads to a peptide-specific T cell response. For instance, they recovered the tumors at day 35, T cells from these tumors could have been tested for their specificity. The authors mention that the spleens of vaccinated mice are larger than in control mice (lines 279-280). What does this according to the authors indicate? Where these CD8+ T cells responding to the peptides injected, and could these spleen derived T cells kill/recognize the tumor cell line in vitro. Such experiments need to be performed.

Minor remarks:

1.At page 7, lines 144-145, the authors state “associated with TAP or proteasome subunit expression defects”. Proteasome defects have not been shown, the word should be removed.

2.In table 3 the authors present their selection of peptides that principally could be presented in the context of HLA-A*0201. They selected 2 peptides with a high score in the prediction software and one peptide because it has a similar score to the earlier found peptide (ppCT16-25) because part of the sequence was also presented in the murine H2-Kb molecule. The latter seem to be an irrelevant argument for the selection of peptides that can bind in HLA-*0201. This makes one wonder about the other peptides with a similar score, as more candidates may actually function as CTL epitope. The authors should give a table showing all these potential candidates.

3.At page 8, line 159, the authors did not use the original MART-1 peptide but the E27L analogue peptide. This should be appropriately mentioned since the original peptide has low binding affinity but good peptide stability.

4.At page 8, line 161, the authors state that based on the half-life of the peptide:MHC complexes (stabilization capacity of the peptides) they have the characteristics of immunogenic peptides. They should refer to the original article about this being Van der Burg et al. J. Immunol. 1996.

5.At page 183 the IGR-Heu cell line, untransfected or transfected with TAP is introduced. The authors should specifically mention that this cell line has a low expression of TAP and provide a reference to their ref 23 for this. Now the reader can’t know that this cell line represents an APM defective tumor cell.

6.At page 10, line 210 it is mentioned “that SPP then generates a 17-aa-long ppCT 9-17 precursor peptide”, this should be “that SPP then generates a 9-aa-long ppCT 9-17 peptide”

7.Figure 4b is labelled as “4c”, please correct.

8.Supplementary fig 3a, please indicate the number of mice used.

9.Based on fig 5a the authors conclude that all peptides are immunogenic but in all fairness the response to peptides 16-25 and 86-100 are low at best. It is advisable to tune down the

conclusion (line 256) on this.

Reviewer #3:
Remarks to the Author:
The authors have previously described a peptide derived from the signal peptide of preprocalcitonin (ppCT) and processed independently from TAP and the proteasome (ref 22, PNAS 2008). In the same paper they showed that ppCT is overexpressed in human lung tumors.
In the current manuscript they describe 3 new peptides from ppCT and studied their processing using inhibitors and siRNA approaches. Finally, they studied the immunogenicity and anti-tumor effect of vaccines against ppCT peptides in two different in vivo models.

Overall the manuscript is not clearly written and the results presented are not convincing, because experiments were not properly designed and lacked crucial controls. Moreover results are often hardly interpretable because of the poor lytic activity observed.

Here are more specific comments:
- identification of new epitopes from ppCT. The authors have previously described an epitope derived from the signal sequence of ppCT (16-25). This epitope was shown to be processed in a TAP-independent and proteasome-independent manner. Here they aim to describe additional epitopes from ppCT. They identified three peptide sequences able to bind HLA-A2, and to stimulate peptide-reactive CD8 T cells from the blood of HLA-A2 cancer patients and healthy donors. Whether these epitopes are naturally processed and presented by cancer cells is not convincingly shown. The authors only obtained short-term polyclonal T-cell lines, and showed a weak lytic activity against the cancer cell lines expressing CT. Because the tumor line is allogeneic and the T cell line is not clonal, the weak killing activity they observed might be due to either non-specific or cross-reactive T cells, different from the T cells that recognize the peptide. This is a classical problem in the field and the literature has described ways to exclude this, the most convincing being the derivation of monospecific CD8 T cell clones.

- the study of the processing of the new peptides suffers from the same limitation. It is based on testing whether inhibition or inactivation of TAP, proteasome, signal-peptide peptidase or ERAD would affect recognition of tumor cells by the CD8 T cells. Except for the previously described peptide ppCT16-25, the results are inconsistent, likely because the signal is very weak (10-20% killing activity at a high E/T ratio) and because the T cell line is not clonal. Positive/negative controls are missing for the effects of the inhibitors, so that it is impossible to conclude whether the effects are specific to the epitope studied (e.g. peptide-pulsed positive controls for cell viability, controls for HLA-A2 expression after SPP inhibition, etc). There are no statistics. The figures show one killing assay repeated in several patients, with weak and inconsistent effects. The conclusions of the authors regarding the processing of these peptides are not supported by the data.

- Notwithstanding the above-comments, from the limited data presented, it would appear that the new peptides are processed in a TAP-dependent manner, and therefore do not qualify as epitopes representative of « immune-escaped tumors ». Only the previously described epitope would qualify. In this regard, the title and the abstract of the manuscript are misleading.

- The authors then perform vaccine studies with ppCT peptides. They induce peptide-specific T cell responses by stimulating human lymphocytes in vitro with the peptides with adjuvant for 2 weeks. They obtain peptide-specific reactivity, and show some cytotoxic activity against their cell line IGR-Heu. However, classical specificity controls are missing, such as e.g. K562 as target cells to exclude NK activity.

- The authors then immunized mice that are transgenic for HLA-A2, and observed anti-peptide

reactivity. Aiming to show the anti-tumor effect of such immunization, they inoculated mice with tumor cells engineered to express HLA-A2 and CT. On days 1, 7 and 21 after tumor inoculation, they immunized mice with the peptide cocktail and poly-IC as an adjuvant. They observed reduced tumor growth in mice receiving the vaccine as compared to mice receiving only DMSO (vehicle control). The problem in that experiment is that the authors cannot exclude that the anti-tumor effect was due to poly-IC, since poly-IC was not present in the vehicle control. Poly-IC is a TLR3 ligand, which is known to exert anti-tumor effects by itself, either directly or by triggering innate immunity mechanisms. Because the vaccine is given after the tumor injection, this is a likely explanation for the results. To exclude this the authors should have poly-IC in their control group.

Additional comments

- Table I: these results are somewhat redundant with similar results published by the authors in 2008 (ref 22). The authors should discuss the rationale to extend the study and integrate the two sets of results. They should also clarify the results, which are expressed in relative terms, by indicating clearly what is the reference for each gene. It appears that for CT the results are expressed relative to normal thyroid, but there is no indication in the manuscript about the origin and number of the human thyroid samples.
- Figure 3: symbols are difficult to read because they are too small

Reviewers' comments:

Reviewer #1: (Remarks to the Author):

The paper by Durgeau et al describes interesting data on T-cell immunity to lung carcinomas. The authors had previously identified a CD8 T-cell epitope from the leader sequence of the preprocalcitonin protein, and showed that this HLA-A2 binding peptide is presented by TAP-negative lung carcinomas. In the current manuscript, they reveal several other T cell epitopes on this protein and demonstrate their dependence on TAP, signal peptidase, signal peptide peptidase and ERAD. Moreover, immunogenicity of these several peptides is tested in lung cancer and healthy PBMC, showing higher CD8 T cell responses in the cancer patients. Finally, mouse models (immunocompetent HLA-A2 transgene and NSG mice) demonstrate the in vivo efficacy of such CD8 T-cell reactivity against tumor growth. This paper encompasses a significant amount of work, is coherent, well written and of major interest due to the TAP-dependent and TAP-independent character of the involved tumor antigens. Immunotherapy will result in selective pressure towards immune-escaped tumors with defects in the antigen processing machinery, and these novel types of TAP-independent neoantigens might constitute a valid approach to counteracting this.

Major issue:

1. In several figures where immunogenicity of the ppCT peptides are portrayed (fig 1, fig 4 and suppl fig 1) the frequencies of IFNg positive CD8 T cells in the cultures are depicted as circles. Do these circles represent repetitive culture wells? Are means depicted (and not median frequencies, as suggested in suppl fig 1)?

In Fig. 1a and the new versions of Fig. 4a and Supplementary Fig. 2b (ex-supplementary Fig 1b), each circle represents the % of ppCT peptide-specific $CD8^+/IFN\gamma^+$ T cells present in the culture of a pool of 8 wells (one column from a 96-well plate). Six to fifteen culture columns were tested for each peptide and each patient. In these figures, as requested by the reviewer below, horizontal lines now correspond to the mean percentages of positive cells in 6-15 pools of 8 culture columns. This is now clearly explained in the figure legends.

In figure 1b and 1d the summary of 15 patients are shown, however the medium values are not coupled to the peptide-stimulated values. Therefore, these graphs are not very insightful. It would be much better if T cell responses are scored for each patient on the basis of a biological relevant cut-off value (e.g. mean of medium condition mean values plus 2 times the SD). Each response that reaches this threshold might be enumerated. Clearly, a lower percentage of responding patients will be observed, but this is a more standardized way to score immunogenicity and spontaneous anti-tumor CD8 T cell responses are usually low in cancer patients. The data from PBMC of healthy donors (suppl fig 1) should be included in the paper itself to directly compare these in figure 1 with those of cancer patients.

As required by the referee, the $CD8^+/IFN\gamma^+$ T-cell response for each patient (total of 15 patients) and healthy donor (total of 12 healthy donors) are now scored on the basis of a biologically relevant cut-off value (e.g. mean of medium condition mean values plus 2 times the SD). Each response that reached this threshold (horizontal dashed line) was enumerated (page 9 of the manuscript), and data are now presented in the new versions of Fig. 1b, Fig. 1c

(ex-Suppl Fig. 1c), Fig. 1e (ex-Fig. 1d) and Fig. 4b. We have also included in the new version of Fig. 1c the data from PBMC of healthy donors (ex-Suppl Fig. 1c).

Minor issues:

2. Introduction: the Trh4 derived TEIPP epitope in the mouse is not derived from the signal peptide, but from the C-terminus of the protein (see reference 36).

2. The sentence has been rewritten and the mistake is now corrected (page 5).

3. Table I: please clarify in the legend of the table how relative expression was calculated. Were delta Ct values of housekeeping genes normalized to healthy thyroid tissue (CALCA) or normal autologous lung (TAPs)? Also, spell out the abbreviations of disease subtypes. The indicated sample numbers are irrelevant for this table.

3. The expression of *CT* was normalized to a pool of 5 human healthy thyroid tissues, and the expression of *TAP* was normalized to autologous healthy lung tissue from each patient. In the legend to Table I, we have now clarified how relative expression was calculated. We have also spelled out the abbreviations and changed the sample numbers in this table so as not to confuse them with the patient numbers used in the figures.

4. Table III: How are Fluorescence Index calculated? What does it mean when these values are lower than 1? This suggests lower HLA-A2 expression than control T2 cells.

4. In Table IV (ex Table III), the fluorescence index (FI) was calculated as follows: FI = (MFI with peptide - MFI without peptide)/MFI without peptide. This is now explained in Table IV legend and Methods (page 22), and a reference is included: (Yang Z et al. J Neurooncol, 2013. 114(1): p. 51-8).
Values lower than 1 mean that the peptide fixation is low. However, the reference is not 1, but 0 (the value obtained with unloaded T2 cells).

5. Suppl fig 3: Explain in legend what HHD-DR3 mice are.

5. HHD-DR3 mice have been explained in Supplementary Fig. 4 (Ex-Supplementary Fig. 3).

6. Fig 5: did vehicle vaccinated mice received DMSO with poly I:C? How many mice were used in fig 5a and how many times were the tumor outgrowth experiments performed?

6. In Fig 5, vehicle-vaccinated mice received DMSO plus poly I:C. This is now corrected in all figures and figure legends (page 41). Five mice per group and per experiment were used, and three experiments were performed. Data correspond to means of the three independent experiments (15 mice). This is now explained in the figure legend (page 41).

Reviewer #2: (Remarks to the Author):

The work of Durgeau et al. revolves around an exciting topic which is the recognition of tumors that in principle may escape immunity by down-regulation of antigen presentation machinery (APM) components, such as TAP. In the last couple of years they have published a series of papers in which they studied the immune response to human preprocalcitonin (CT), a self-antigen that was spontaneously recognized by CTL from a lung cancer patient. These CTL preferentially recognized tumor cells with a low TAP expression. The current manuscript presents their data on their study of the response to this CT self-antigen in many patients as well as provides the first evidence that representative peptides may be used as immunotherapeutic vaccine to control tumor growth.

Major remarks

1. In Tables 1 and 2 they present the data on CT and TAP RNA expression and CT protein expression. At the RNA level 55% (8/14) of the ADC tumor overexpress CT, at the protein level this is only 20%. In SCC 3/6 show overexpression at the RNA level, this is 0% at the protein level. This discrepancy is not explained nor the consequence for their statement that 75% of the lung tumors expressed low levels of TAP1 and TAP2 mRNA. At this point one rather would like to see protein data on TAP, so that a better estimation of the real percentage of tumors with TAP defects can be made.

1. In Table I, we present data on *CT* and *TAP* RNA expression for 28 NSCLC tumour samples, and in Table II, we present data on CT protein expression for 215 distinct NSCLC tumour samples. The discordance in our RNA and protein expression levels arose from the fact that RNA expression was performed on a cohort of fresh tumours normalized to autologous "normal" tissue samples, while protein expression was performed on an independent cohort of FFPE tumour samples. Moreover, RT-PCR analysis is much more sensitive than immunohistochemistry (IHC). From these RT-PCR data, we stated that around 71% of the lung tumours expressed low levels of *TAP1* and/or *TAP2* mRNA; 20 out of 28 NSCLC tumours (in bold and italics in Table I). This is now explained in Table I, legend.

As required by the referee, and to better estimate the true percentage of tumours with TAP defects, we have included protein data on TAP2 expression for 135 FFPE lung tumour samples. These data are now included in Table III, and examples of anti-TAP2 IHC are shown in Supplementary Fig. 1b. These data indicate that only 14% of lung tumour samples display high (normal) expression levels of TAP2. These data are also included in the manuscript (pages 7 and 8).

2. In figure 1 the responses of IFNgamma producing CD8+ T cells from lung cancer patients as measured by flow cytometry is shown. The data presentation and interpretation is unclear and the reader is left with several questions:

a) What is the definition of a positive reaction;

b) while the M&M section state that per peptide tested one column of a 96-well plate is tested (8 wells) the graphs clearly show more data points for some peptides and less data points for others, suggesting that multiple tests have been combined whereas in other conditions have been left out;

c) the PBMC were stimulated for 7 days and then restimulated for the flow cytometric analysis, it is common to compare in such cultures the medium (background production control) and the peptide stimulated cells from the same culture. Currently, it is hard to tell if the IFNg positive CD8 T cells all responded to the peptide during the test. These data should be presented by the authors, furthermore, the authors should provide one flow cytometry example of such a test in the supplementary data.

2. In figure 1,
a) A positive reaction is defined by a significant increase, as determined by the Mann-Whitney U test, in IFNγ production following PBMC stimulation with a given peptide as compared to PBMC stimulated with medium containing a vehicle control alone (DMSO). As requested by referee 1, we have now added a biologically relevant cut-off value (e.g. mean of the medium condition mean values plus 2 times the SD) as a threshold for significance. Data are now included in the new versions of Fig. 1b, Fig. 1c, Fig. 1e and Fig. 4b, and cut-off values are represented by horizontal dashed lines. We also indicated in figure 1, legend, results from the Mann-Whitney U test (* $p < 0.05$; ** $p < 0.001$; ***, $p<0.0001$).

b) In this figure, PBMC were stimulated in 96-well plates with indicated peptides. Cells from 8 wells (1 column) were pooled and then stained with anti-CD8 mAb and anti-IFNγ mAb. For each ppCT peptide, 12-15 columns were included. For media and control peptides, a half 96-well plate (6 columns) was used for some patients for whom insufficient amounts of PBMCs were available. Each circle corresponds to a pool of cells from 8 wells. This is now explained in Fig. 1 legend (page 38).

c) The PBMC were stimulated for 14 days with each peptide or with medium (containing DMSO alone), and then restimulated for 6 h in the same conditions for flow cytometry analysis. These data include responding $CD8^+$/$IFN\gamma^+$ T cells from 6-15 replicates (1 replicate is presented by one circle, which corresponds to data from 8 wells), and the mean percentages of $CD8^+$/$IFN\gamma^+$ T cells are shown (horizontal lines). This is now explained in Fig. 1, legend.
To distinguish responding $CD8^+$/$IFN\gamma^+$ T cells from non-responding $CD8^+$ T cells, we now provide flow cytometry examples of such a test in supplementary data (novel version of Suppl Fig. 2a).

3. Figure 1b shows the accumulated data of 15 patients. The authors should define what a positive response is and then present a bar graph of the number of responding patients to each peptide. Now their conclusion (page 8, lines 171-173) is hard to follow.

3. As requested by reviewers 1 and 2, we have now included a new version of Fig. 1b, where T-cell responses are scored on the basis of a biologically relevant cut-off value (e.g. mean of medium condition mean values plus 2 times the SD), and each response that reaches this threshold (horizontal dashed line) was considered positive and enumerated (page 9). Results from this figure and Fig. 1c (healthy donors) are now summarized in a histogram (supplementary Fig. 2c).

4. To show that the CD8+ T cells responsive to the ppCT peptides can recognize naturally processed peptides presented at the surface of tumor cells, the data in figure 2 is presented. Isolated CD8+ T cells from 3 patients are used. Overall there is only a low level of cytotoxicity shown, generally lower than 20% at the highest E:T ratio. I would consider this

suggestive but certainly not definitive proof. The authors should use a more sensitive read-out, such as IFNg production by the T cells upon recognition of the tumour cells. Preferably, the authors should isolate T cell clones from their cultures to make this point.

4. To demonstrate that the ppCT-derived peptides are naturally processed peptides and presented on the surface of tumour cells, we have now included in Fig. 2a cytotoxicity data from $CD8^+$ T cells generated from 3 different patients (patients 1, 3 and 13) and, in Fig. 2c, IFNγ release from T-cell clones or cloids derived from patient 1 (Heu) and stimulated with the autologous tumour cell lines IGR-Heu and IGR-Heu-TAP.

5. To show the effect of TAP expression, figure 2 also shows the killing of tumor cells that overexpress TAP. Again, while the data is suggestive, the differences in killing between TAP+, wild type and control cells are marginal. T-cell clones to the identified peptides are required to provide final proof.

5. To show the effect of TAP expression we have now included in Fig. 2a cytotoxicity data from $CD8^+$ T cells generated from 3 patients (patients 1, 3 and 13) toward IGR-Heu and IGR-Heu-TAP established from patient 1, and in Fig. 2c, IFNγ release by T-cell clones and cloids derived from patient 1 and stimulated with autologous tumour cells (IGR-Heu and IGR-Heu-TAP).

6. In figure 4, the immunogenicity of peptides is tested using patient's PBMC. Several questions come up:
a) Two longer peptides ppCT1-15 and ppCT86-100 are used. As the authors defined an epitope as ppCT9-17, it is not clear to me why they chose to make a long peptide missing the last two amino acids of this epitope. This should be explained, or rather a ppCT1-17 peptide should be used;
b) page 11, line 239 states "from most NSCLC patients" but only five are shown;
c) a discrepancy exists between the data shown for patient 3 in fig 4a (positive response to ppCT1-15) and fig4b (no response to this peptide;
d) fig 4b, patient 20 shows a response to the ppCT peptide cocktail but not to the individual peptides, this is strange as at least one peptide of the cocktail should be positive.

6. In Figure 4,
a) We chose the ppCT1-15 peptide because it includes at least 3 additional potential epitopes, of 9 aa ($ppCT_{7-15}$, $ppCT_{4-12}$ and $ppCT_{2-10}$), predicted by SYFPEITHI algorithm to bind to HLA-A2. This is now included in Supplementary Table 1 and is explained in the manuscript (page 12).

b) "from most NSCLC patients": this sentence has been rewritten, and for more accuracy, the number of responding patients among those tested is included (7 out of 10 and 5 out of 10 NSCLC patients; see page 12).

c) The discrepancy between the data shown for patient 3 in Fig. 4a and 4b (Fig. 4a and 4c in the new version of the figure) is due to a mistake in patient numbering. Indeed, data presented in Fig. 4a are not from patient 3, but from patient 2. We regret this mistake, which is now corrected.

d) In the previous version of Fig. 4b, patient 20 showed a response to the ppCT peptide cocktail, but not to the individual peptides, possibly because we were limited in the number of available PBMCs for this patient, and therefore we used less cells (10^5 instead of $2x10^5$) for this experiment. To avoid any concern, we have repeated this experiment with an additional patient (patient 51) for whom we had enough cells, and more convincing results have now been obtained and are included in this figure (novel version of Fig. 4c).

7. In figure 5 data is shown on their experiments in two different mouse models. These experiments can be considered essential. In the tumour control experiments (figures 5c and 5f) they show vaccinated mice or mice injected with vehicle only. It is important to realize that vaccination comprises the injection of peptide with poly I:C, whereas the vehicle control is DMSO only. The proper control to show that the tumour control is mediated by peptide-induced T cells and not by the adjuvant is to also inject the control group with the adjuvant (poly I:C).
In figure 5c they use the Lewis cancer cell line LL2 as a target tumour. Previously it has been published (Shime et al. PNAS 2012, p2066) that poly I:C injection controls tumour outgrowth of Lewis cancer cells via the repolarization of tumour associated macrophages. This effectively means that currently there is no formal proof that the vaccine-activated T cells control tumour outgrowth in this experiment. Also the increase in CD8 T cells in the tumour can be an indirect effect of changes in TAMs.

7. In Figs. 5c and 5f, the vehicle control included both DMSO and poly I:C. To avoid confusion, this is now fully written in the manuscript, figures and the figure legend. We also included in Supplementary Fig. 4b experiments indicating that DMSO alone (vehicle) or combined with poly I:C (vehicle + adjuvant) did not induce any IFNγ response. In contrast, the $ppCT_{50-59}$ peptide delivered with poly I:C together with DMSO induced an IFNγ response.

In Fig. 5c, we used D122-HHD-ppCT, an LL2 cell line transgenic for HLA-A2 and transfected with human ppCT, as a tumour target. As mentioned above, all negative controls included both DMSO and poly (I:C). Because poly(I:C) combined with DMSO was unable to induce an anti-tumour response, this proves that the observed effect was due to the ppCT-based cancer vaccine and not to poly(I:C). Moreover, a CD8 T-cell cytotoxic response toward ppCT-expressing tumour cells was demonstrated *ex vivo* and was inhibited by anti-MHC class I mAb (Supplementary Fig. 4c).

8. With respect to the data presented in figure 5f/g where NSG mice are injected with human PBMC and the IGR-heu tumor, the same arguments apply. Furthermore, as the authors state that the injected PBMC after vaccination control tumor outgrowth, they should also show that vaccination leads to a peptide-specific T cell response. For instance, they recovered the tumors at day 35, T cells from these tumors could have been tested for their specificity. The authors mention that the spleens of vaccinated mice are larger than in control mice (lines 279-280). What does this according to the authors indicate? Where these CD8+ T cells responding to the peptides injected, and could these spleen derived T cells kill/recognize the tumor cell line in vitro. Such experiments need to be performed.

8. In figure 5f/g, vehicle control included both poly(I:C) and DMSO. This was corrected in the manuscript, figures and figure legends.

As requested by the reviewer, in order to support the observation that vaccination of NSG mice after injection of human PBMC controls tumour growth, we assessed the peptide-specific T-cell response in TIL from vaccinated and non-vaccinated (DMSO plus poly(I:C) control) mice. Results indicated an increase in the absolute number of human $CD8^+$ T cells producing IFNγ when restimulated *ex vivo* with the peptide cocktail in TIL from vaccinated mice as compared to non-vaccinated (DMSO plus poly(i:c) control) mice (Fig. 5h). Therefore, we also deleted the sentence regarding the spleens of vaccinated and non-vaccinated mice.

Minor remarks:

1. At page 7, lines 144-145, the authors state "associated with TAP or proteasome subunit expression defects". Proteasome defects have not been shown, the word should be removed.

1. The sentence on page 7 has now been removed.

2. In table 3 the authors present their selection of peptides that principally could be presented in the context of HLA-A*0201. They selected 2 peptides with a high score in the prediction software and one peptide because it has a similar score to the earlier found peptide (ppCT16-25) because part of the sequence was also presented in the murine H2-Kb molecule. The latter seem to be an irrelevant argument for the selection of peptides that can bind in HLA-*0201. This makes one wonder about the other peptides with a similar score, as more candidates may actually function as CTL epitope. The authors should give a table showing all these potential candidates.

2. We have removed the sentences related to the $ppCT_{91-100}$ epitope and explained that this epitope was actually selected because it has a higher fluorescence index and better immunogenicity than other peptides displaying similar or higher predictive scores. As requested by the reviewer, we have now added to Supplementary Table I all potential HLA-*0201-restricted epitopes, and we explain how we chose the selected ones (page 8). This was based not only on their predictive scores, but also on their capacity to effectively bind to HLA-A2 molecules using the T2 cell line test, and to induce a CD8 T-cell response.

3. At page 8, line 159, the authors did not use the original MART-1 peptide but the E27L analogue peptide. This should be appropriately mentioned since the original peptide has low binding affinity but good peptide stability.

3. On page 8 we specify that the MART-1 peptide used as a positive control was the E27L analogue peptide, and we have included the corresponding references.

4. At page 8, line 161, the authors state that based on the half-life of the peptide:MHC complexes (stabilization capacity of the peptides), they have the characteristics of immunogenic peptides. They should refer to the original article about this being Van der Burg et al. J. Immunol. 1996.

4. On page 8, we referred to the original article (Van der Burg et al. J. Immunol. 1996) concerning characteristics of immunogenic peptides.

5. At page 183 the IGR-Heu cell line, untransfected or transfected with TAP is introduced. The authors should specifically mention that this cell line has a low expression of TAP and provide a reference to their ref 23 for this. Now the reader can't know that this cell line represents an APM defective tumour cell.

5. We specified that the IGR-Heu cell line displays weak expression of TAP and we have provided a reference (Durgeau et al J Immunol 2011), page 9.

6. At page 10, line 210 it is mentioned "that SPP then generates a 17-aa-long ppCT 9-17 precursor peptide", this should be "that SPP then generates a 9-aa-long ppCT 9-17 peptide"

6. On page 10, we have corrected the sentence so that it is more easily understood (pages 11 and 12 in the new version of the manuscript).

7. Figure 4b is labelled as "4c", please correct.

7. We have changed Figure 4c to Figure 4b.

8. Supplementary fig 3a, please indicate the number of mice used.

8. In supplementary Fig 3a, (Supplementary Fig. 4a in the new version of the manuscript), we used 5 mice per group and presented three independent experiments (15 mice per group in total).

9. Based on fig 5a the authors conclude that all peptides are immunogenic but in all fairness the response to peptides 16-25 and 86-100 are low at best. It is advisable to tune down the conclusion (line 256) on this.

9. The conclusion pertained to Fig. 5a, and the immunogenicity of the ppCT peptides has now been tuned down (page 13).

Reviewer #3: (Remarks to the Author):

The authors have previously described a peptide derived from the signal peptide of preprocalcitonin (ppCT) and processed independently from TAP and the proteasome (ref 22, PNAS 2008). In the same paper they showed that ppCT is overexpressed in human lung tumors. In the current manuscript they describe 3 new peptides from ppCT and studied their processing using inhibitors and siRNA approaches. Finally, they studied the immunogenicity and anti-tumor effect of vaccines against ppCT peptides in two different in vivo models.

Overall the manuscript is not clearly written and the results presented are not convincing, because experiments were not properly designed and lacked crucial controls. Moreover, results are often hardly interpretable because of the poor lytic activity observed.

Here are more **specific comments**:
- identification of new epitopes from ppCT. The authors have previously described an epitope derived from the signal sequence of ppCT (16-25). This epitope was shown to be processed in a TAP-independent and proteasome-independent manner. Here they aim to describe additional epitopes from ppCT. They identified three peptide sequences able to bind HLA-A2, and to stimulate peptide-reactive CD8 T cells from the blood of HLA-A2 cancer patients and healthy donors. Whether these epitopes are naturally processed and presented by cancer cells is not convincingly shown. The authors only obtained short-term polyclonal T-cell lines, and showed a weak lytic activity against the cancer cell lines expressing CT. Because the tumor line is allogeneic and the T cell line is not clonal, the weak killing activity they observed might be due to either non-specific or cross-reactive T cells, different from the T cells that recognize the peptide. This is a classical problem in the field and the literature has described ways to exclude this, the most convincing being the derivation of monospecific CD8 T cell clones.

- Cytotoxic activity data were obtained from enriched T-cell lines toward the ppCT-expressing IGR-Heu tumour cell line established from patient 1 (Heu). Weak lytic activity toward cancer cells expressing low levels of MHC-I molecules (see Supplementary Fig. 4e) is common, even with autologous CTL clones. Notably, among the enriched T-cell lines generated here, those established from patient 1 (Heu) were used in cytotoxicity experiments toward the autologous tumour cell lines IGR-Heu and IGR-Heu-TAP. This is now mentioned in the manuscript (page 9). Moreover, killing of IGR-Heu tumour cells was inhibited by neutralizing anti-MHC class I mAb (W6/32), further supporting the conclusion that it is specific and TCR-mediated (see Fig. 2a).

We also performed, for the three patients, experiments with the EBV-B-cell line, established from patient 1 (Heu-EBV), unloaded and loaded with each of the ppCT peptides. Results indicated that peptide-pulsed EBV-B cells were efficiently killed by the enriched T-cell lines, as opposed to unloaded target cells, which were only weakly lysed (see Fig. 2b).
In addition, we performed cytotoxicity experiments toward “NK” target cell line K562. Results indicated that none of the generated CD8 T cells were able to kill K562 target cells, supporting the conclusion that the cytotoxic activity toward IGR-Heu tumour cells is specific and does not correspond to “NK-like” activity.

Finally, as requested by the reviewer, we have now established, from patient 1, T-cell clones or cloids specific to each of the peptides. Results included in the new version of Fig. 2c

indicate that T cells specific to $ppCT_{9-17}$, $ppCT_{50-59}$ and $ppCT_{91-100}$ peptides produced higher levels of IFNγ when stimulated with the autologous IGR-Heu-TAP cell line than when stimulated with IGR-Heu. In contrast, T-cell clones generated from the same patient toward the $ppCT_{16-25}$ epitope produced higher levels of IFNγ when stimulated with IGR-Heu than when stimulated with IGR-Heu-TAP. These results further support the conclusion that these peptides are naturally processed by TAP-dependent and -independent pathways, respectively (see page 10 of the manuscript).

- the study of the processing of the new peptides suffers from the same limitation. It is based on testing whether inhibition or inactivation of TAP, proteasome, signal-peptide peptidase or ERAD would affect recognition of tumor cells by the CD8 T cells. Except for the previously described peptide ppCT16-25, the results are inconsistent, likely because the signal is very weak (10-20% killing activity at a high E/T ratio) and because the T cell line is not clonal.

Positive/negative controls are missing for the effects of the inhibitors, so that it is impossible to conclude whether the effects are specific to the epitope studied (e.g. peptide-pulsed positive controls for cell viability, controls for HLA-A2 expression after SPP inhibition, etc).
There are no statistics. The figures show one killing assay repeated in several patients, with weak and inconsistent effects. The conclusions of the authors regarding the processing of these peptides are not supported by the data.

- As requested by the reviewer, we also performed experiments on the processing of the four peptides with the T-cell clones and cloids generated for each peptide from NSCLC patient 1 (new version of Fig. 3b). Results clearly indicated that, while epoxomicin inhibits IFNγ production by $ppCT_{9-17}$-, $ppCT_{50-59}$- and $ppCT_{91-100}$-specific T cells, it has no effect on cytokine production by $ppCT_{16-25}$-specific T cells. In contrast, DCI inhibited IFNγ production by all epitope-specific T cells, further supporting the conclusion that cleavage of the ppCT signal peptide is a prerequisite for further processing of the protein precursor. Results included in figure 3c further support the involvement of SP in the processing of all ppCT peptides, while those presented in Figures 3d and 3e support the involvement of SPP in the processing of only $ppCT_{9-17}$ and $ppCT_{16-25}$, and ERAD in the processing of $ppCT_{50-59}$- and $ppCT_{91-100}$ epitopes, respectively (see pages 11 and 12 of the manuscript).

In addition, we have included all controls indicating that the chemical inhibitors and siRNA had no or only weak effects on cell viability (Supplementary Figure 3c) and HLA-A2 molecule expression levels on IGR-Heu and IGR-Heu-TAP target cells (Supplementary Figure 3d).

Finally, we have included statistics.

- Notwithstanding the above-comments, from the limited data presented, it would appear that the new peptides are processed in a TAP-dependent manner, and therefore do not qualify as epitopes representative of « immune-escaped tumors ». Only the previously described epitope would qualify. In this regard, the title and the abstract of the manuscript are misleading.

- In this manuscript, we not only describe new ppCT peptides that are processed via a TAP/proteasome-dependent mechanism, but also a ppCT peptide cocktail that includes both TAP-dependent and -independent epitopes, as well as 15-aa long peptides, and that, when

injected together with an adjuvant into two mouse models, is able to control growth of «immune-escaped tumours » and tumours that may express variable levels of TAP. The title and the abstract of our manuscript summarize all our *in vitro* and *in vivo* findings on the ppCT tumour antigen and the derived peptide-based cancer vaccine.

- The authors then perform vaccine studies with ppCT peptides. They induce peptide-specific T cell responses by stimulating human lymphocytes in vitro with the peptides with adjuvant for 2 weeks. They obtain peptide-specific reactivity, and show some cytotoxic activity against their cell line IGR-Heu. However, classical specificity controls are missing, such as e.g. K562 as target cells to exclude NK activity.

- Cytotoxicity toward the NK target cell line K562 was performed and is now included in Supplementary Fig. 4c. Results indicated that none of the peptide-specific CTL mediated cytotoxic activity toward K562 target cells. Moreover, cytotoxicity toward IGR-Heu tumor cells was inhibited by anti-MHC-class I mAb W6/32, further supporting the conclusion that cytotoxicity is MHC-class-I-restricted and TCR-mediated (Supplementary Fig. 4c). As mentioned above, we also used K562 to exclude NK-like cytotoxicity toward human T lymphocytes stimulated *in vitro* with the peptides (no adjuvant is needed in these experiments); see Figure 2b.

- The authors then immunized mice that are transgenic for HLA-A2, and observed anti-peptide reactivity. Aiming to show the anti-tumor effect of such immunization, they inoculated mice with tumor cells engineered to express HLA-A2 and CT. On days 1, 7 and 21 after tumor inoculation, they immunized mice with the peptide cocktail and poly-IC as an adjuvant. They observed reduced tumor growth in mice receiving the vaccine as compared to mice receiving only DMSO (vehicle control). The problem in that experiment is that the authors cannot exclude that the anti-tumor effect was due to poly-IC, since poly-IC was not present in the vehicle control. Poly-IC is a TLR3 ligand, which is known to exert anti-tumor effects by itself, either directly or by triggering innate immunity mechanisms. Because the vaccine is given after the tumor injection, this is a likely explanation for the results. To exclude this the authors should have poly-IC in their control group.

- In all our *in vivo* experiments, vehicle control included both DMSO and poly(I:C), since each one alone had no effect. This is now corrected in the manuscript, figures and figure legends. To avoid confusion, we used vehicle for DMSO alone and vehicle plus adjuvant for DMSO + poly(I:C). A supplementary Figure 4b that includes DMSO alone (vehicle), DMSO plus poly(I:C) control (vehicle + adjuvant), and the most immunogenic peptide $ppCT_{50\text{-}59}$ administered with DMSO plus poly(I:C) is also now included.

Additional comments

- Table I: these results are somewhat redundant with similar results published by the authors in 2008 (ref 22). The authors should discuss the rationale to extend the study and integrate the two sets of results. They should also clarify the results, which are expressed in relative terms, by indicating clearly what is the reference for each gene. It appears that for CT the results are expressed relative to normal thyroid, but there is no indication in the manuscript about the origin and number of the human thyroid samples.

- In Table I, none of the included samples had ever been previously published. As requested by the reviewer, we now explain the rationale for extending the study (page 7). We have also clarified the reference for each gene (*CALCA* and *TAP*) and indicated the origin and number of the human thyroid samples in Table I, legend, as well as the corresponding reference.

- Figure 3: symbols are difficult to read because they are too small.

- In Fig. 3, we have increased the size of all symbols and we use colours to distinguish the different patients. This has also been done for all the other figures.

Reviewers' Comments:

Reviewer #1:
Remarks to the Author:
The authors did a fine job to substantiate their initial data. The paper is now extended with novel results supporting their original message. The manuscript improved extensively.

Reviewer #2:
Remarks to the Author:
The authors have done a tremendous job in answering my questions. All answers and changes have been satisfactory.

Reviewer #3:
Remarks to the Author:
The manuscript has increased in readability and a number of issues have been addressed by the authors. There remain, however, a number of issues of serious concern.

1. Key to the demonstration that the new antigens are naturally processed and presented is the ability to show that not only CTL lines but also monospecific CTL clones can recognize tumor cells. The authors have added new data in the manuscript showing recognition of tumors by CTL clones or cloids (Figure 2c). However, they do not indicate for which antigen they used clones and for which antigen they used cloids. It appears from the rebuttal letter that they only used a clone for the previously identified antigen ppCT16-25, and used cloids for the newly described antigens. Moreover, they do not describe what they mean by « cloid ». Cited reference #63 is not contributive in that respect because it only talks about actual clones. If the « cloids » result from failed attempts to clone (and therefore could also be called pseudoclones), then they do not qualify as acceptable reagents to demonstrate the natural processing and presentation of the new antigens. Because these antigens form the basis of the manuscript, this issue should be solved. It may well be, however, that it is hard/impossible to derive CTL clones against these antigens. In this case, the authors can use the alternative approach, which is to do the lysis assays with the non-clonal CTL line and add an excess of competing unlabelled target cells pulsed with the peptide of interest. If killing of the tumor cells is specifically directed against the peptide, it should be fully abrogated in the presence of an excess of peptide-pulsed cold targets.

2. The fact the peptide ppCT9-17 is TAP-dependent is intriguing and potentially interesting, but not convincingly demonstrated. Indeed, opposite results are shown on Fig 2a/b and on Fig 2c regarding respective recognition of IGR-Heu and IGR-Heu-TAP. Fig S3c also is not consistent for the 2 patients. Perhaps this is linked to the fact that IGR-Heu is not completely null for TAP. The authors may obtain more convincing results using TAP inhibitors such as ICP47, which can be (co)-transfected and completely inactivate TAP function. CRISPR-Cas9 for TAP would also be acceptable but would need more work.

3. Fig S4c: if it is true the ppCT9-17 peptide is TAP-dependent, then it is unclear why the authors only tested ppCT9-17-induced CTL on TAP-negative targets IGR-Heu and not on IGR-Heu-TAP cells.

4. Fig 1abc (and Fig S2): the legend is still unclear here. Please indicate clearly in the legend (as detailed in the reply to reviewer #2) that PBMC were stimulated for 2 weeks with the peptide prior to the 6h-stimulation for the intracellular IFN staining (this point is not clear in the M&M because it comes in 2 separate sections).

5. Figure 3: authors should indicate how many times the experiments were repeated.

Response to Reviewers' comments:

Re: NCOMMS-17-03346.
Title: "Human preprocalcitonin self-antigen generates proteasome/TAP-dependent and -independent epitopes triggering optimised T-cell responses toward immune-escaped tumours », by Aurélie Durgeau et al.

Reviewer #3:

The manuscript has increased in readability and a number of issues have been addressed by the authors. There remain, however, a number of issues of serious concern.

1. Key to the demonstration that the new antigens are naturally processed and presented is the ability to show that not only CTL lines but also monospecific CTL clones can recognize tumor cells. The authors have added new data in the manuscript showing recognition of tumors by CTL clones or cloids (Figure 2c). However, they do not indicate for which antigen they used clones and for which antigen they used cloids. It appears from the rebuttal letter that they only used a clone for the previously identified antigen ppCT16-25, and used cloids for the newly described antigens. Moreover, they do not describe what they mean by « cloid ». Cited reference #63 is not contributive in that respect because it only talks about actual clones. If the « cloids » result from failed attempts to clone (and therefore could also be called pseudoclones), then they do not qualify as acceptable reagents to demonstrate the natural processing and presentation of the new antigens. Because these antigens form the basis of the manuscript, this issue should be solved.

It may well be, however, that it is hard/impossible to derive CTL clones against these antigens. In this case, the authors can use the alternative approach, which is to do the lysis assays with the non-clonal CTL line and add an excess of competing unlabelled target cells pulsed with the peptide of interest. If killing of the tumor cells is specifically directed against the peptide, it should be fully abrogated in the presence of an excess of peptide-pulsed cold targets.

1. To demonstrate that the ppCT antigenic peptides are naturally processed and presented, we used 3 T-cell clones for the $ppCT_{16-25}$ epitope: the previously described T-cell clone Heu161 and 2 new T-cell clones (Heu57 and Heu143) generated from patient 1 (Heu). For the $ppCT_{9-17}$, $ppCT_{50-59}$ and $ppCT_{91-100}$ epitopes, we used 4 T-cell cloids for each (enriched T-cell lines cloned at 10-20 cells/well). This is now explained in the manuscript and two additional references were included (Echchakir H, et al., Int Immunol. 2000 Apr;12(4):537-46 and Dudley ME, Nishimura MI, Holt AK, Rosenberg SA. J Immunother. 1999 Jul;22(4):288-98).

To further demonstrate the natural processing and presentation of the $ppCT_{9-17}$, $ppCT_{50-59}$ and $ppCT_{91-100}$ antigenic peptides, we used the approach suggested by the reviewer by performing cytotoxicity experiments with the enriched CTL lines toward the IGR-Heu-TAP tumour cell line and adding an excess of competing unlabeled IGR-Heu-TAP target cells pulsed with the peptide of interest ($ppCT_{9-17}$, $ppCT_{50-59}$ or $ppCT_{91-100}$). Indeed, the IGR-Heu cell line expresses very low levels of HLA-A2 molecules (Supplementary Fig. 4e), and therefore it is

impossible to load it with external synthetic peptides. Results indicated that killing of Cr^{51}-labeled tumour cells is abrogated in the presence of an excess of peptide-pulsed cold target cells (Supplementary Fig. 3a), supporting the conclusion that the cytotoxic activity is specifically directed against the peptide of interested.

2. The fact the peptide ppCT9-17 is TAP-dependent is intriguing and potentially interesting, but not convincingly demonstrated. Indeed, opposite results are shown on Fig 2a/b and on Fig 2c regarding respective recognition of IGR-Heu and IGR-Heu-TAP. Fig S3c also is not consistent for the 2 patients. Perhaps this is linked to the fact that IGR-Heu is not completely null for TAP. The authors may obtain more convincing results using TAP inhibitors such as ICP47, which can be (co)-transfected and completely inactivate TAP function. CRISPR-Cas9 for TAP would also be acceptable but would need more work.

As the reviewer probably knows, signal-anchor sequences direct proteins to the endoplasmic reticulum (ER) membrane and can anchor the protein in a type II or type I orientation. A type I anchor has its N-terminal region in the ER and a type II anchor has its N-terminal region in the cytoplasm.

[Redacted]

We have previously reported that the ppCT signal peptide has a type II orientation i.e. signal sequence spanning the ER membrane with the n region exposed towards the cytosol and the c region facing the ER lumen (El Hage F et al., Ann N Y Acad Sci. 2013. 1283:75-80; El Hage F et al., Proc Natl Acad Sci U S A. 2008 Jul 22;105(29):10119-24).

ppCT signal peptide fragments are released into the ER lumen and presented to CTL by MHC-I molecules in a TAP-independent manner (ppCT$_{16-25}$) or into the cytosol where they are processed by the proteasome (ppCT$_{1-17}$). The resulting fragment (ppCT$_{9-17}$) is transported by TAP into the ER lumen, where it binds to HLA-A2 molecules and then conveyed to the cell surface to be recognized by CTL.

Indeed, after cleavage by SP and SPP, a ppCT$_{1-17}$ signal peptide fragment (Red) is released into the cytoplasm to be processed by the proteasome-TAP pathway , while the ppCT$_{16-25}$ peptide is released into the ER where it is presented by a TAP-independent pathway (Blue). Thus, the ppCT$_{9-17}$ epitope is processed by the proteasome/TAP pathway. It might also be generated from the ppCT$_{1-17}$ signal peptide fragment by a proteasome-independent mechanism involving cytoplasmic proteasomes, and then transported into the ER in a TAP-dependent fashion.

[Redacted]

Overall, the fact that the ppCT$_{9-17}$ epitope is TAP-dependent is not that intriguing but rather required for presentation to CTL by MHC-I molecules on the tumour cell surface.

As mentioned by the reviewer, the IGR-Heu cell line is not completely null for TAP expression. Indeed, we previously demonstrated that this tumour cell line, which express two identified tumour antigens, i.e. the mutated α-actinin-4 antigen (Echchakir H et al., Cancer Res. 2001 61(10):4078-83) and the ppCT antigen (El Hage F et al., Proc Natl Acad Sci U S A. 2008 105(29):10119-24.), is able to process the $actn4_{91–100}$ neoepitope in a TAP-dependent manner to be recognized by the Heu171 T-cell clone and the $ppCT_{16-25}$ epitope in TAP-independent manner to be recognized by the Heu161 T-cell clone (**see Fig. R3-3**). Moreover, while IFNγ, which enhances MHC-I and TAP expression, optimizes $actn4_{91–100}$ epitope presentation, it inhibits $ppCT_{16-25}$ epitope presentation (Durgeau A et al., J Immunol. 2011 187(11):5532-9).

[Redacted]

The opposite results shown in Fig. 2a/b and Fig. 2c regarding the respective recognition of IGR-Heu and IGR-Heu-TAP might be linked to the fact that we used polyclonal T-cell lines in figure 2a/b or because the IGR-Heu cell line is not completely null for TAP. Indeed the expression of TAP in IGR-Heu tumour cells is sufficient to permit processing and presentation of the TAP-dependent mutated $actn4_{91–100}$ epitope and the $ppCT_{9-17}$ epitope.

As requested by the referee, we used siRNA targeting TAP1, which inhibit TAP expression in IGR-Heu without affecting the expression of HLA-A2 molecules (see Supplementary Fig. 3f and g). Results indicated that siRNA-TAP1 had no effect on recognition by anti-$ppCT_{9-17}$ $CD8^+$ T cells. Indeed, the expression of TAP in IGR-Heu cells is already low, and further inhibition had no effect.

[Redacted]

3. Fig S4c: the ppCT9-17 peptide is TAP-dependent, then it is unclear why the authors only tested ppCT9-17-induced CTL on TAP-negative targets IGR-Heu and not on IGR-Heu-TAP cells.

3. In Fig. S4c, we tested ppCT9-17-induced CTL toward TAP-positive (IGR-Heu-TAP) target cells. The figure is now corrected.

4. Fig 1abc (and Fig S2): the legend is still unclear here. Please indicate clearly in the legend (as detailed in the reply to reviewer #2) that PBMC were stimulated for 2 weeks with the peptide prior to the 6h-stimulation for the intracellular IFN staining (this point is not clear in the M&M because it comes in 2 separate sections).

4. For Fig. 1abc and Fig. S2, we added additional information to make the legend clearer. As requested by the reviewer, we added that the PBMC were stimulated for 14 days with each peptide or with medium (containing DMSO alone), and then restimulated for 6 h in the same conditions for flow cytometry analysis with anti-CD8 and anti-IFNγ.

5. Figure 3: authors should indicate how many times the experiments were repeated.

5. These experiments were performed 3 times. This is now indicated in the figure legend.

Reviewers' Comments:

Reviewer #3:
Remarks to the Author:
My comments are attached and were included in red within the authors' rebuttal letter.

Re: NCOMMS-17-03346.
Title: “Human preprocalcitonin self-antigen generates proteasome/TAP-dependent and -independent epitopes triggering optimised T-cell responses toward immune-escaped tumours », by Aurélie Durgeau et al.

Reviewer #3:

2. The fact the peptide ppCT9-17 is TAP-dependent is intriguing and potentially interesting,

2. A TAP-dependent processing of ppCT9-17 could indeed make sense. It would be useful for the average reader to elaborate on this and explain in the discussion why it makes sense, for a type II signal anchor like this one, to have a TAP-dependent cytosolic processing from the N-terminal part of the signal sequence (9-17) and a TAP-independent ER processing for the C-terminal part of the signal (16-25).
Yet, the authors should be cautious in their conclusion, as the TAP-dependency of ppCT9-17 is suggested but not demonstrated in the manuscript (see below).

2. As suggested by the reviewer, we explained in the discussion why it makes sense, for a type II signal anchor like ppCT leader sequence, to have a TAP-dependent cytosolic processing from the N- terminal part of the signal sequence (9-17) and a TAP-independent ER processing for the C- terminal part of the signal (16-25) and included a reference (Martoglio B, Dobberstein B. Trends Cell Biol. 1998. 8(10):410-5). See page 16, lines 360-366.
We also attenuated our conclusion regarding TAP-dependency of ppCT9-17 throughout the manuscript.

3. In Fig. S4c, we tested ppCT9-17-induced CTL toward TAP-positive (IGR-Heu-TAP) target cells. The figure is now corrected.

The figure was not corrected. The panels were inverted, but they still show the same data. The recognition of IGR-Heu-TAP by ppCT9-17 CTL is still not shown. Only shown for that peptide is the recognition of IGR-Heu cells. This is not acceptable.

In all the figures we used the same order: ppCT9-17, ppCT50-59, ppCT91-100 and ppCT-16-25. We placed the pp16-25 peptide at the end to distinguish this TAP-independent epitope for the other epitopes. But, in the previous version of figure 4c, we made a mistake by placing the ppCT epitopes in increasing order as we did in the X-axis of Figure 1 (ppCT9-17, ppCT-16-25, ppCT50-59 and ppCT91-100).

We did not change the panel, but we just corrected its placement in the figure by using the same order than the other figures: ppCT9-17, ppCT50-59, ppCT91-100 and ppCT-16-25. Indeed, the cytotoxicity of ppCT9-17-induced CTL shown in the original figure was performed toward IGR-Heu-TAP and not IGR-Heu. We are sorry for this mistake. We now used the same symbols than ppCT50-59 and ppCT91-100 in a new version of Supplementary Figure 4C, and included in Figure S3-4 for the reviewer the results toward IGR-Heu cells. In this figure, lower cytotoxic activities toward IGR-Heu tumour cells were observed (Fig. S3-4; see below).

[Redacted]

Reviewer #3:

2. The fact the peptide ppCT9-17 is TAP-dependent is intriguing and potentially interesting, but not convincingly demonstrated.

2. A TAP-dependent processing of ppCT9-17 could indeed make sense. It would be useful for the average reader to elaborate on this and explain in the discussion why it makes sense, for a type II signal anchor like this one, to have a TAP-dependent cytosolic processing from the N-terminal part of the signal sequence (9-17) and a TAP-independent ER processing for the C-terminal part of the signal (16-25).
Yet, the authors should be cautious in their conclusion, as the TAP-dependency of ppCT9-17 is suggested but not demonstrated in the manuscript (see below).

2. As suggested by the reviewer, we explained in the discussion why it makes sense, for a type II signal anchor like ppCT leader sequence, to have a TAP-dependent cytosolic processing from the N- terminal part of the signal sequence (9-17) and a TAP-independent ER processing for the C- terminal part of the signal (16-25) and included a reference (Martoglio B, Dobberstein B. Trends Cell Biol. 1998). See page 16, lines 355-366.
We also attenuated our conclusion regarding TAP-dependency of ppCT9-17 throughout the manuscript (page 16, lines 352 and 359).

- This makes little sense: if the killing of IGR-Heu by ppCT9-17 is indeed TAP-dependent, and if it is observed despite already low TAP expression by IGR-Heu, then further reduction of TAP expression should reduce recognition by ppCT9-17 CD8 T cells. So the argument of the authors is wrong here, and the data do not support the TAP-dependency of ppCT9-17.

- As mentioned by the reviewer, our previous data do not support the TAP-dependency of ppCT9-17 epitope. Consequently, to further evaluate whether the killing of IGR-Heu tumour cells by ppCT9-17-specific CTL is TAP-dependent, despite the already low TAP expression by IGR-Heu, we performed additional cytotoxicity experiments toward IGR-Heu tumour cells, untreated or pre-treated with siRNA targeting TAP1, using 20 10^6 PBMC from 6 NSCLC patients stimulated *in vitro* with the ppCT9-17 peptide. Data shown in Supplementary Fig. 4b & c (ex. Supplem Fig. 3d & e) from 2 representative patient PBMC indicated that further reduction of TAP expression in IGR-Heu tumour cells resulted in a decrease in recognition by ppCT9-17-specific CD8 T cells. These more convincing data now support the conclusion that the processing of the ppCT9-17 epitope is TAP-dependent.

- Along the same line, the opposite results between Fig 2a/b and 2c remain unexplained and inconsistent with the authors' statement. The argument that Fig2a/b used polyclonal T-cell lines does not hold, since the authors now show in Fig S3a that 99% of the killing activity of the polyclonal lines is specific. Moreover, Fig. 2c did not use clones for ppCT9-17, but cloids, which are also polyclonal.

- Similarly, we performed new experiments with 20 10^6 PBMC from 2 additional NSCLC patients stimulated *in vitro* with the peptide ppCT9-17 peptide. Results shown in the new version of Figure 2a (ex. Fig. 2a & b) are concordant with those presented in Fig. 2b (ex. Fig.

2c) and Supplem Fig. 3b (ex Supplem Fig. 3a), further supporting the conclusion that processing of the ppCT9-17 epitope is likely TAP-dependent.

- Lastly, Fig S4c, which the authors failed to amend as requested (see below), is also not consistent with TAP-dependent recognition of ppCT9-17, as the authors did not test recognition of IGR-Heu-TAP cells but only TAP-negative cells IGR-Heu cells.

- Lastly, we repeated cytotoxicity experiments with ppCT peptide-specific $CD8^+$ T cells from spleens of vaccinated HHD-DR3 mice toward IGR-Heu and IGR-Heu-TAP, preincubated in medium or in the presence of neutralizing anti-MHC-I mAb W6/32, and K562 target cells. Supplem Fig. 5c (ex. Fig S4c) shows that IGR-Heu-TAP target cells are more strongly killed by ppCT9-17-specific CTL as compared to TAP-low IGR-Heu cells, consistent with TAP-dependent recognition of ppCT9-17 epitope.

- In the absence of more convincing data, the authors should be much more cautious in their conclusion about TAP-dependency of peptide ppCT9-17.

Using higher numbers of PBMC from additional NSCLC patients, and IGR-Heu and IGR-Heu-TAP target cells previously checked for TAP expression, we now present more convincing and consistent results showing the TAP-dependency of ppCT9-17 peptide.

3. In Fig. S4c, we tested ppCT9-17-induced CTL toward TAP-positive (IGR-Heu-TAP) target cells. The figure is now corrected.

- This is not true. The figure was not corrected. The panels were inverted, but they still show the same data. The recognition of IGR-Heu-TAP by ppCT9-17 CTL is still not shown. Only shown for that peptide is the recognition of IGR-Heu cells. This is not acceptable.

- We did not change the panel corresponding to killing by ppCT9-17-specific CD8 T cells because the assembly of the original version of Figure 4c was not correct. Indeed, the target presented in this original figure was IGR-Heu-TAP and not IGR-Heu. In this experiment we performed cytotoxicity toward both IGR-Heu and IGR-Heu-TAP. Therefore, we added in Fig. R3-4 for the reviewer results from this cytotoxicity experiment for both IGR-Heu and IGR-Heu-TAP cells (see below). However, because of this error, we decided to repeat these experiments for ppCT9-17, ppCT50-59 and ppCT91-100 peptides, and new results are now included in Supplem 5c (ex. Supplem 4c). In these figures (Fig. R3-4 and Supplem 5c), lower cytotoxic activities toward IGR-Heu tumour cells than IGR-Heu-TAP cells were observed for ppCT9-17-specific CTL.

[Redacted]

Figure S3-4. Cytotoxic activity of $ppCT_{9-17}$ peptide-specific $CD8^+$ T cells from spleens of vaccinated HHD-DR3 mice. Cytotoxicity of $ppCT_{9-17}$-specific CTL toward IGR-Heu and IGR-Heu-TAP tumour cells, preincubated in medium or in the presence of neutralizing anti-MHC-I mAb W6/32, and K562 target cells was determined one week after the last immunization by a conventional 4-h ^{51}Cr release assay. E:T ratios are indicated. Data shown correspond to one of 3 independent experiments.

Reviewers' Comments:

Reviewer #3:

Remarks to the Author:

The authors have corrected the mistakes and provided more convincing evidence about the TAP-dependency of peptide ppCT9-17. The revised discussion is more balanced.
