# Supplementary material for: Human preprocalcitonin self-antigen generates TAP-dependent and -independent epitopes triggering optimised T-cell responses toward immune-escaped tumours
Source: Nat Commun. 2018 Nov 30;9:5097. doi: 10.1038/s41467-018-07603-1 (PMC6269466; doi:10.1038/s41467-018-07603-1)
Supplement: Supplementary file 1 — Peer Review File [file 41467_2018_7603_MOESM1_ESM.pdf]

Supplementary Information

Human preprocalcitonin self-antigen generates TAP-dependent and -independent epitopes triggering optimised T-cell responses toward immune-escaped tumours,

Durgeau et al.,

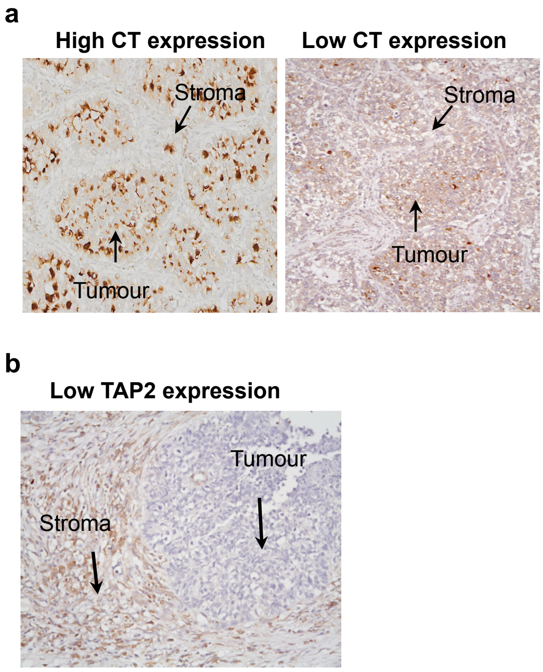

Supplementary Figure 1: Expression of CT and TAP2 in NSCLC tumours. **a.** Expression of CT in NSCLC tumours. Tumour sections from 215 early-stage NSCLC patients were stained with anti-CT mAb. Representative images of immunostaining from two tumours are shown. **b.** Expression of TAP2 in NSCLC tumours. Tumour sections from 135 early-stage NSCLC patients were stained with anti-TAP2 mAb. One representative image of immunostaining is shown. CT: calcitonin.

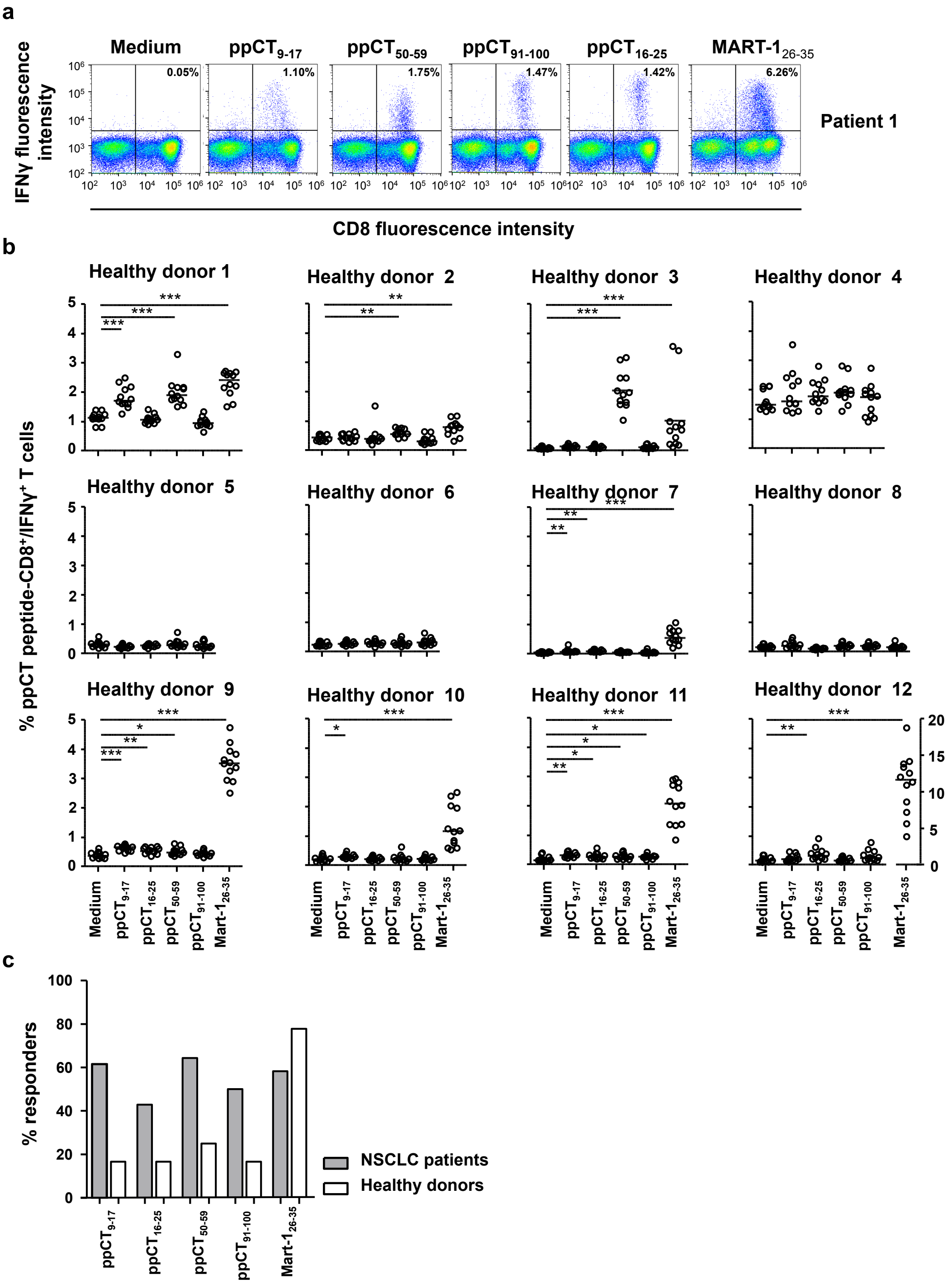

Supplementary Figure 2: Immunogenicity of ppCT-derived peptides in human PBMC. **a.** Cytoplasmic expression of IFNγ in $CD8^+$ T cells, as determined by intracellular immunofluorescence. PBMC from patient 1 were stimulated for 14 days in 96-well plates with indicated peptides or with medium (containing DMSO alone), then restimulated for 6 h in the same conditions, and then cells from eight wells were pooled and labelled with anti-CD8 mAb; after membrane permeabilization, cells were stained with anti-IFNγ. DMSO was used as a negative control and the highly immunogenic HLA-A2.1-restricted MART-$1_{26\text{-}35}$ peptide served as a positive control. **b.** Immunogenicity of ppCT-derived peptides in healthy donor PBMC. PBMC were stimulated in 96-well plates with indicated peptides and then cells from eight wells were pooled and labelled with anti-CD8; after membrane permeabilization, cells were stained with anti-IFNγ mAb. Samples were analysed using an Accuri C6 cytometer and data were processed by Cflow software. Horizontal bars correspond to the mean percentages of positive cells from eight culture wells. *, $p<0.05$; **, $p<0.01$; ***, $p<0.001$ (two-tailed Mann-Whitney U test). **c.** Percentages of NSCLC and healthy donors responding to ppCT peptides. The percentages of patients (among the 15 tested) and healthy donors (among the 12 tested) responding to each one of the ppCT peptides were determined from results shown in Fig. 1b and Fig. 1c, respectively.

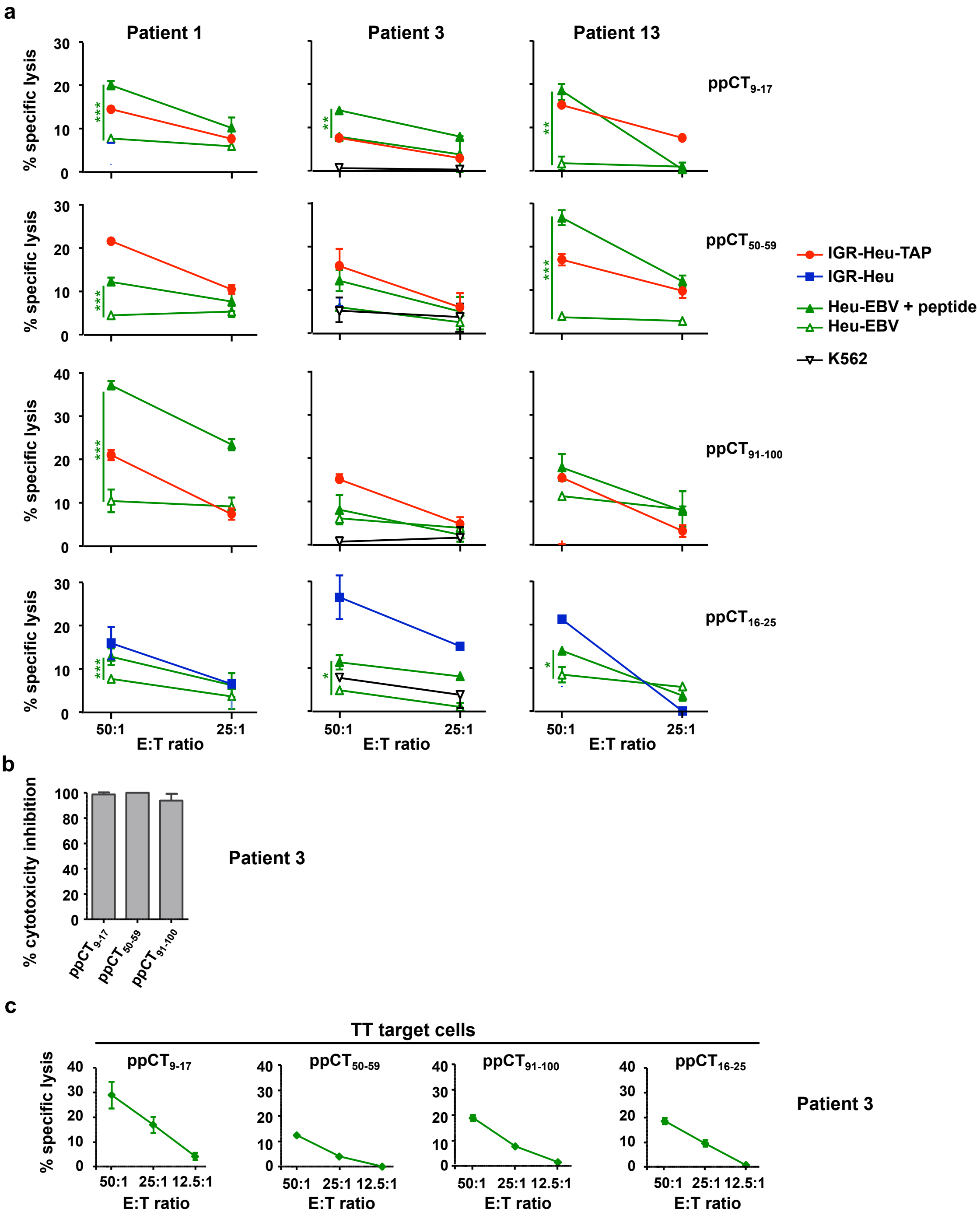

Supplementary Figure 3: Specificity of ppCT-peptide-stimulated CD8^{+} T cells. NSCLC patient PBMC were stimulated *in vitro* with indicated peptides, and then CD8^{+} T cells were isolated and their cytotoxic activity tested. **a.** The EBV-B cell line (Heu-EBV), unpulsed or pulsed with the specific peptide, and K562 were used as targets. IGR-Heu or IGR-Heu-TAP tumour cells were included as controls. Cytotoxicity was determined by a conventional 4-h chromium (^{51}Cr) release assay at indicated E:T ratios. Each value corresponds to mean (± SD) of percentages of lysis from triplicates. Data shown represent experiments from 3 patient's PBMC. *, p<0.05; **, p<0.01; ***, p<0.001 (two-tailed Student's unpaired t test). Red: IGR-Heu-TAP, bleu: IGR-Heu, and green: Heu-EBV targets. **b.** Inhibition of cytotoxic activity of ppCT peptide-specific CD8^{+} T cells toward IGR-Heu-TAP tumour cells by adding unlabeled peptide-pulsed target cells. Cytotoxicity was performed toward Cr^{51}-labeled IGR-Heu-TAP tumour cells in the absence or presence of an excess of competing unlabeled IGR-Heu-TAP cells pulsed with the indicated ppCT peptides. Percentages of cytotoxicity inhibition were determined using the formula [(% lysis without peptide - % lysis with peptide) / % lysis without peptide] x 100[1]. Results are means (± SD) of percentages of lysis from triplicates of three distinct T-cell lines. **c.** Cytotoxicity of ppCT peptide-specific CD8^{+} T cells toward the allogeneic ppCT^{+} MTC cell line, TT. NSCLC patient 3 PBMC were stimulated *in vitro* with indicated peptides, and then CD8^{+} T cells were isolated and their cytotoxic activity toward the TT cell line was tested. Results are means (± SD) of percentages of lysis from triplicates. Green: Patient 3. E:T: effector:target. SD: standard deviation.

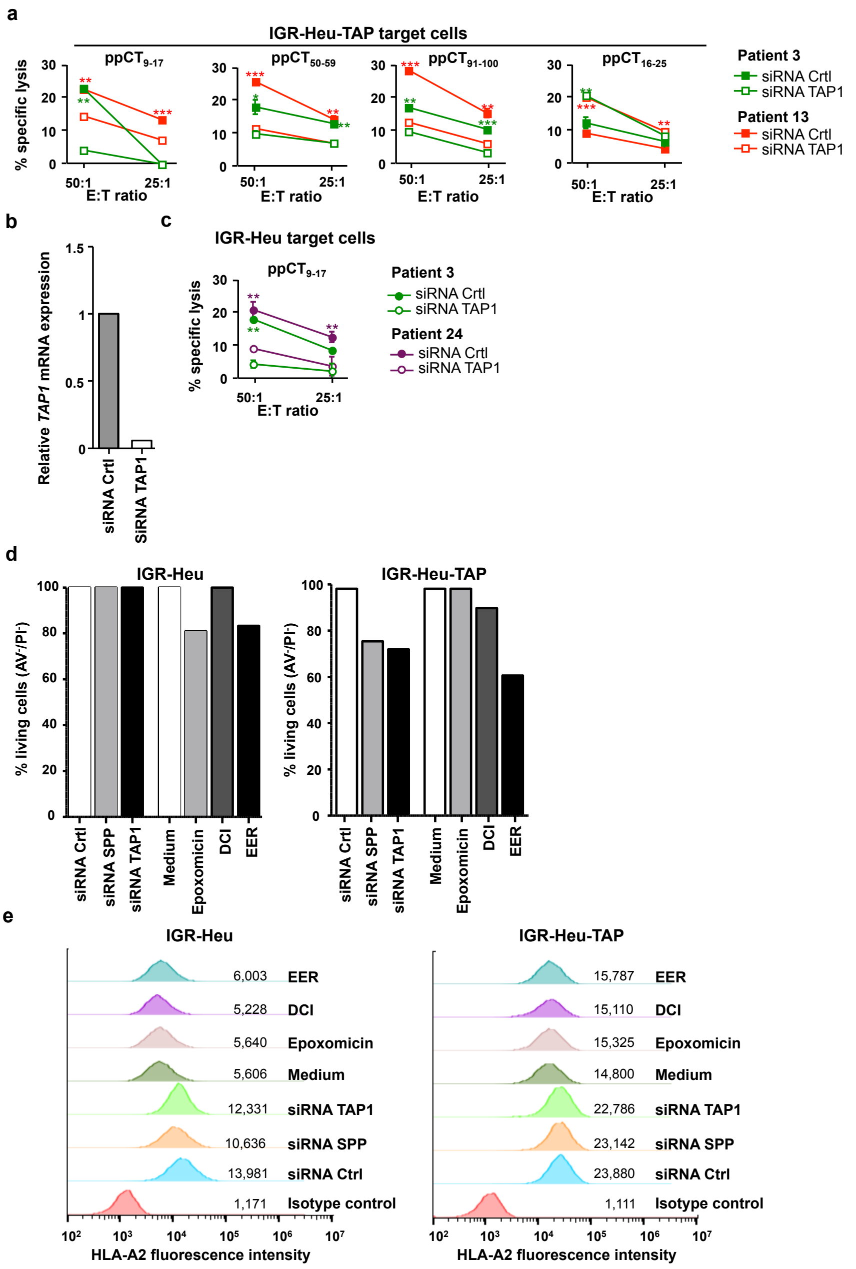

Supplementary Figure 4: Cytotoxicity of ppCT-specific T cells and effects of inhibitors and siRNA on cell viability and HLA-A2 expression. **a.** Cytotoxic activity of ppCT peptide-stimulated CD8$^+$ T cells toward IGR-Heu-TAP tumour cells. PBMC from NSCLC patients 3 and 13 were stimulated *in vitro* with indicated peptides, and then CD8$^+$ T cells were isolated and cytotoxicity toward IGR-Heu-TAP tumour cells electroporated with siRNA TAP1 or siRNA Crtl was tested. Results are means (± SD) of percentages of lysis from triplicates. *, p<0.05; **, p<0.01; ***, p<0.001 (two-tailed Student's unpaired t test). **b.** RT-PCR analysis of *TAP1* expression in IGR-Heu tumour cells electroporated or not with siRNA TAP1 or siRNA Crtl. **c.** Downregulation of TAP expression in IGR-Heu cells inhibits recognition by anti-ppCT$_{9-17}$ CD8$^+$ T cells. PBMC from patients 3 and 24 were stimulated *in vitro* with the ppCT$_{9-17}$ peptide, and then CD8$^+$ T cells were isolated and cytotoxicity toward IGR-Heu tumour cells, untreated or pretreated with siRNA TAP1 or siRNA Crtl, was determined. Data shown are from 2 independent experiments out of 6. **d.** Treatment of IGR-Heu and IGR-Heu-TAP cell lines with siRNA or with inhibitors has weak effects on cell viability. IGR-Heu (left) and IGR-Heu-TAP (right) tumour cells were electroporated with siRNA targeting SPP or TAP1, or treated with epoxomicin, DCI or EER, and living cells were then detected by flow cytometry using annexin V and propidium iodide (AV/PI) labelling. siRNA control and medium alone were used as negative controls. Percentages of living cells (AV$^-$/PI$^-$) are shown. **e.** Treatment of IGR-Heu and IGR-Heu-TAP cell lines with siRNA or with inhibitors does not alter HLA-A2 surface expression levels. IGR-Heu (left) and IGR-Heu-TAP (right) tumour cells were electroporated with siRNA targeting SPP or TAP1, or treated with epoxomicin, DCI or EER and then were analysed for HLA-A2 molecule surface expression by flow cytometry. Numbers correspond to mean fluorescence intensity (MFI). DCI: dichloroisocoumarin, EER1: eeyarestatin 1. Green: patient 3, red: patient 13, pink: patient 24.

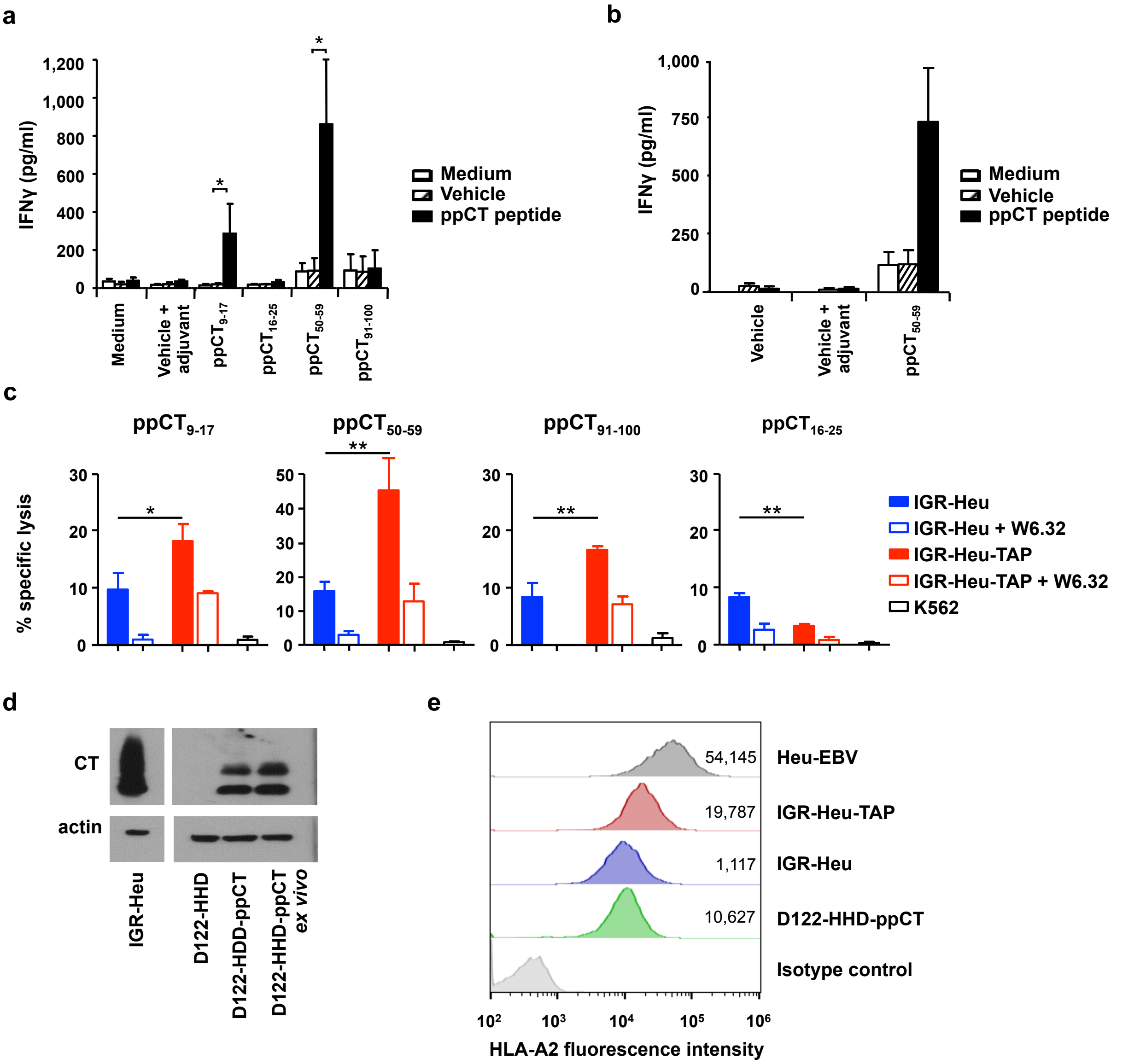

Supplementary Figure 5: Immunogenicity of ppCT-derived peptides *in vivo*. **a.** Immunogenicity of the ppCT-derived peptide in HHD-DR3 mice. Three-to-five per group HHD-DR3 mice (humanized for both HLA-A2 and HLA-DR3, and β2m$^{-/-}$, H-2Db$^{-/-}$, IAβ$^{-/-}$, IAα$^{-/-}$ and IEβ$^{-/-}$) were immunized four times at a one-week interval with each ppCT-derived peptide co-administered with poly (I:C) or with poly (I:C) plus DMSO control. One week after the final immunization, spleens were recovered and cells were cultured in medium or restimulated with each peptide; next, IFNγ secretion was measured by ELISA. **b.** DMSO and poly(I:C) plus DMSO had no effect on IFNγ production by immunized mice. HHD-DR3 mice were injected four times at a one-week interval with DMSO, poly(I:C) plus DMSO or ppCT$_{50-59}$ co-administered with vehicle plus adjuvant. One week after the final immunization, spleens were recovered and cells were cultured in medium or restimulated with DMSO or ppCT$_{50-59}$ peptide emulsified in DMSO, and then IFNγ secretion was measured. Results are means (± SEM) of three independent experiments (n=12). **c.** Cytotoxic activity of ppCT peptide-specific CD8^{+} T cells from spleens of vaccinated mice. Cytotoxicity of ppCT$_{9-17}$-, ppCT$_{50-59}$-, ppCT$_{91-100}$- and ppCT$_{16-25}$-specific CTL toward IGR-Heu and IGR-Heu-TAP, preincubated in medium or in the presence of neutralizing anti-MHC-I mAb W6/32, and K562 target cells was determined 1 week after the last immunization by a conventional 4-h ^{51}Cr release assay. E:T ratio, 50:1. Results are means (± SD) of percentages of lysis from triplicates. *, $p<0.05$; **, $p<0.01$ (two-tailed Student's unpaired t test). Data shown correspond to one of 3 independent experiments. **d.** ppCT protein expression in D122-HHD-ppCT murine lung tumours. D122-HHD tumour cells were infected with a human ppCT-encoding lentivirus (rLV/hCT); then, ppCT expression was analysed by western blot using specific mAb (Dako). Tumours recovered from HHD-DR3 mice engrafted with D122-HHD-ppCT cells (D122-HHD-ppCT *ex vivo*) were included. Non-transduced D122-HHD and IGR-Heu tumour cell lines were used as a negative and positive control, respectively. **e.** Surface expression of HLA-A2 molecules on Heu-EBV, IGR-Heu-TAP, IGR-Heu and D122-HHD-ppCT target cells. Mean fluorescence intensity (MFI) are indicated. CT: calcitonin.

Supplementary Table 1: HLA-A2-restricted ppCT-derived peptides predicted by SYFPEITHI algorithm

10-aa long peptides

**Start position**	**End position**	**aa sequence**	**SYFPEITHI score**	**FI**
9	***18***	***FLALSILVLL***	***29***	***0.19***
14	23	ILVLLQAGSL	24	
**50**	**59**	**LLAALVQDYV**	**24**	**2.06**
17	26	LLQAGSLHAA	22	
41	***50***	***TLSEDEARLL***	***22***	***0.19***
11	20	ALSILVLLQA	21	
40	49	ATLSEDEARL	20	
125	134	DLERDHRPHV	19	
**16**	**25**	**VLLQAGSLHA**	**18**	**0.12**
22	31	SLHAAPFRSA	18	
45	54	DEARLLLAAL	18	
48	57	RLLLAALVQD	18	
49	58	LLLAALVQDY	18	
104	113	HTFPQTAIGV	18	
53	62	ALVQDYVQMK	16	0.03
87	***96***	***NLSTCMLGTY***	***16***	***0.02***
13	22	SILVLLQAGS	15	
23	32	LHAAPFRSAL	15	
31	40	ALESSPADPA	15	
5	*14*	*KFSPFLALSI*	*14*	*0.04*
6	*15*	*FSPFLALSIL*	*14*	*0.01*
8	17	PFLALSILVL	14	
52	61	AALVQDYVQM	14	
68	77	QEQEREGSSL	14	
7	16	SPFLALSILV	13	
10	19	LALSILVLLQ	13	
33	42	ESSPADPATL	13	
42	51	LSEDEARLLL	13	
43	52	SEDEARLLLA	13	
46	55	EARLLLAALV	13	
54	63	LVQDYVQMKA	13	
1	*10*	*MGFQKFSPFL*	*12*	*0.03*
30	39	SALESSPADP	12	
79	88	SPRSKRCGNL	12	
**91**	**100**	**CMLGTYTQDF**	**12**	**0.13**
110	119	AIGVGAPGKK	12	
3	*12*	*FQKFSPFLAL*	*11*	*0.04*
57	66	DYVQMKASEL	11	
60	69	QMKASELEQE	11	
76	85	SLDSPRSKRC	11	
96	105	YTQDFNKFHT	11	0.02

9-aa long peptides

**Start position**	**End position**	**aa sequence**	**SYFPEITHI score**	**FI**
**9**	**17**	**FLALSILVL**	**28**	**0.85**
10	18	LALSILVLL	24	
41	***49***	***TLSEDEARL***	***24***	***1.78***
15	23	LVLLQAGSL	20	
24	32	HAAPFRSAL	20	
49	57	LLLAALVQD	20	
17	25	LLQAGSLHA	19	
46	54	EARLLLAAL	19	
50	58	LLAALVQDY	19	
58	66	YVQMKASEL	19	0.01
11	19	ALSILVLLQ	18	
13	21	SILVLLQAG	18	
51	59	LAALVQDYV	18	
53	61	ALVQDYVQM	18	
7	*15*	*SPFLALSIL*	*17*	
47	55	ARLLLAALV	17	
14	22	ILVLLQAGS	16	
18	26	LQAGSLHAA	16	
34	42	SSPADPATL	16	
87	*95*	*NLSTCMLGT*	*16*	
43	51	SEDEARLLL	15	
76	84	SLDSPRSKR	15	
4	*12*	*QKFSPFLAL*	*14*	
110	118	AIGVGAPGK	14	
126	134	LERDHRPHV	14	
2	*10*	*GFQKFSPFL*	*13*	
12	20	LSILVLLQA	13	
16	24	VLLQAGSLH	13	
31	39	ALESSPADP	13	
48	56	RLLLAALVQ	13	
85	93	CGNLSTCML	13	
92	*100*	*MLGTYTQDF*	*13*	
108	116	QTAIGVGAP	13	
6	*14*	*FSPFLALSI*	*12*	
22	30	SLHAAPFRS	12	
30	38	SALESSPAD	12	
42	50	LSEDEARLL	12	
45	53	DEARlLLAA	12	
54	62	LVQDYVQMK	12	
91	*99*	*CMLGTYTQD*	*12*	
114	122	GAPGKKRDM	12	
118	126	KKRDMSSDL	12	

FI (fluorescence index) = mean fluorescence intensity (MFI) with the given peptide - MFI without peptide / MFI without peptide. Selected peptides are in bold. Peptides with high predictive score and low binding affinity toward HLA-A*0201 or low immunogenicity are in bold and italic. Peptides with high predictive scores included in $ppCT_{1-15}$ and $ppCT_{86-100}$ are in italic.

Supplementary Reference

1. Peterson, M.E. & Long, E.O. Inhibitory receptor signaling via tyrosine phosphorylation of the adaptor Crk. *Immunity* 29, 578-588 (2008).

a

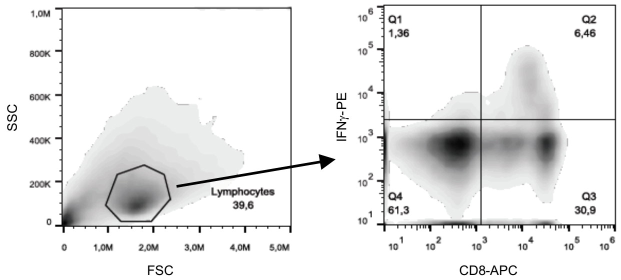

b

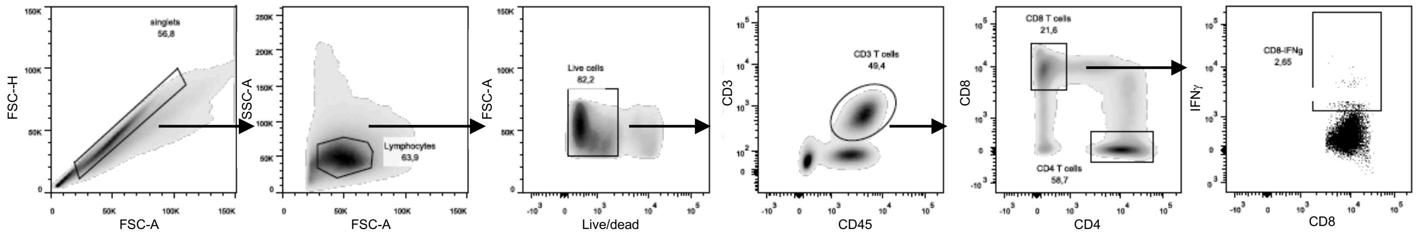

Supplementary Method: Gating strategy for immunostaining. **a.** Gating strategy for *in vitro* experiments. The percentage of $CD8^{+}IFN\gamma^{+}$ cells was obtained after debris were removed by FSC/SSC gating. **b.** Gating strategy for *in vivo* experiments. Singlets were selected by FSC-A/FSC-H gating, then debris and dead cells were removed by FSC-A/SSC-A and Live/dead/FSC-A gating, respectively. $CD8^{+}$ T cells were selected among double positive cells in CD45/CD3 gating, and the percentage of $IFN\gamma^{+}$ T cells among $CD8^{+}$ T cells was given.
